# A *Clostridium difficile*-Specific, Gel-Forming Protein Required for Optimal Spore Germination

**DOI:** 10.1128/mBio.02085-16

**Published:** 2017-01-17

**Authors:** M. Lauren Donnelly, William Li, Yong-qing Li, Lauren Hinkel, Peter Setlow, Aimee Shen

**Affiliations:** a Department of Microbiology and Molecular Genetics, University of Vermont, Burlington, Vermont, USA; b Department of Physics, East Carolina University, Greenville, North Carolina, USA; c Program in Cellular, Molecular & Biomedical Sciences, University of Vermont, Burlington, Vermont, USA; d Department of Molecular Biology and Biophysics, University of Connecticut Health Center, Farmington, Connecticut, USA; e Department of Molecular Biology and Microbiology, Tufts University Medical School, Boston, Massachusetts, USA; The University of Oklahoma Health Sciences Center

## Abstract

*Clostridium difficile* is a Gram-positive spore-forming obligate anaerobe that is a leading cause of antibiotic-associated diarrhea worldwide. In order for *C. difficile* to initiate infection, its aerotolerant spore form must germinate in the gut of mammalian hosts. While almost all spore-forming organisms use transmembrane germinant receptors to trigger germination, *C. difficile* uses the pseudoprotease CspC to sense bile salt germinants. CspC activates the related subtilisin-like protease CspB, which then proteolytically activates the cortex hydrolase SleC. Activated SleC degrades the protective spore cortex layer, a step that is essential for germination to proceed. Since CspC incorporation into spores also depends on CspA, a related pseudoprotease domain, Csp family proteins play a critical role in germination. However, how Csps are incorporated into spores remains unknown. In this study, we demonstrate that incorporation of the CspC, CspB, and CspA germination regulators into spores depends on CD0311 (renamed GerG), a previously uncharacterized hypothetical protein. The reduced levels of Csps in *gerG* spores correlate with reduced responsiveness to bile salt germinants and increased germination heterogeneity in single-spore germination assays. Interestingly, asparagine-rich repeat sequences in GerG’s central region facilitate spontaneous gel formation *in vitro* even though they are dispensable for GerG-mediated control of germination. Since GerG is found exclusively in *C. difficile*, our results suggest that exploiting GerG function could represent a promising avenue for developing *C. difficile*-specific anti-infective therapies.

## IMPORTANCE

The spore-forming bacterium *Clostridium difficile* is a leading cause of health care-associated infections. While a subset of antibiotics can treat *C. difficile* infections (CDIs), the primary determinant of CDI disease susceptibility is prior antibiotic exposure, since it reduces the colonization resistance conferred by a diverse microflora. Thus, therapies that minimize perturbations to the gut microbiome should be more effective at reducing CDIs and their recurrence, the main source of disease complications. Given that spore germination is essential for *C. difficile* to initiate infection and that *C. difficile* uses a unique pathway to initiate germination, methods that inhibit distinct elements of germination could selectively prevent *C. difficile* disease recurrence. Here, we identify GerG as a *C. difficile*-specific protein that controls the incorporation of germinant signaling proteins into spores. Since *gerG* mutant spores exhibit germination defects and are less responsive to germinant, GerG may represent a promising target for developing therapeutics against CDI.

## INTRODUCTION

The spore-forming bacterium *Clostridium difficile* is a leading cause of health care-associated infections and gastroenteritis-associated death worldwide (1, 2). *C. difficile* infections also occur in community and commercial farm settings (3–5). The Centers for Disease Control and Prevention in the United States recently designated *C. difficile* an urgent threat to the health care system in part because antibiotic usage is a major risk factor for *C. difficile* infection (6). Antibiotic treatment eliminates members of the gut microflora that normally suppress *C. difficile* vegetative growth and thus increases patient susceptibility to *C. difficile* infections (7–9). Antibiotic usage may also promote *C. difficile* spore germination by altering the balance of microbially generated bile salt germinants. These changes may effectively increase the infectious dose of *C. difficile* (8, 10–12), since spores are the primary infectious form of this obligate anaerobe (13).

During infection, *C. difficile* spores germinate upon sensing select bile salts in the mammalian gastrointestinal tract (10, 14); the germinating spores then transform into the toxin-secreting vegetative cells that cause disease (7). A key step during this process is the degradation of the spore cortex, a thick protective layer of modified peptidoglycan (15, 16). The cortex is critical to maintaining spore dormancy, since it keeps the spore core, which contains the cell’s genetic material, in a partially dehydrated state that minimizes cellular metabolism. Removal of this physical constraint by cortex hydrolases allows the core to fully hydrate, resume metabolism, and initiate outgrowth (15, 16).

Interestingly, the signaling process that controls cortex hydrolysis in *C. difficile* exhibits marked differences from the other spore-forming bacteria studied to date (17). While most spore formers use inner membrane-bound germinant receptors to trigger germination in response to amino acids and sugars (17–19), *C. difficile* lacks these receptors and instead uses the CspC pseudoprotease as a germinant receptor to sense bile acids (20). When *C. difficile* CspC senses germinant, it activates the subtilisin-like serine protease CspB to remove an inhibitory propeptide from the cortex hydrolase SleC (21). Activation of the SleC zymogen then allows the hydrolase to degrade the thick protective cortex layer (22, 23). Cortex degradation subsequently induces the release of calcium dipicolinic acid (CaDPA) from the spore core (23), which leads to full hydration of the core (23, 24).

In the model organism *Bacillus subtilis*, the order of cortex hydrolysis and CaDPA release is reversed: germinant binding induces CaDPA release, and CaDPA directly activates the cortex hydrolase CwlJ (16). While there is little homology in the germination signaling proteins used by *B. subtilis* relative to *C. difficile* (17–19), many *C. difficile* germination regulators have homologs in *Clostridium perfringens* (17, 18). The functions of these regulators, however, differ between the two clostridial organisms. In *C. perfringens*, Csp proteins are encoded as individual proteases that are functionally interchangeable, with any one of the Csps, CspA, CspB, and CspC, being able to proteolytically activate the SleC zymogen (25, 26). In contrast, *C. difficile* CspB and CspA are produced as a fusion protein that undergoes interdomain processing during sporulation (21, 27). Furthermore, both CspA and CspC harbor catalytic site mutations that render them inactive such that CspB is the only Csp that can proteolytically activate SleC (21). Interestingly, *C. difficile* CspA controls the incorporation of the CspC germinant receptor into mature spores (27), indicating that CspC and CspA have divergent functions in *C. difficile* relative to *C. perfringens*.

Since the CspA and CspC pseudoproteases are conserved only in *C. difficile*’s closest relatives in the *Peptostreptococcaceae* family (27, 28), *C. difficile*’s germinant signaling pathway appears to be unique relative to other organisms studied to date (17, 23). In support of this notion, we recently identified the lipoprotein GerS as another key germination regulator specific to the *Peptostreptococcaceae* family (22). GerS is necessary for SleC activity but is dispensable for pro-SleC cleavage. In this report, we identify a novel *C. difficile-*specific protein, CD0311, that is required for optimal *C. difficile* spore germination. Using a variety of functional assays, we determine the stage of germination impaired by loss of CD0311, herein referred to as GerG, and analyze its potential functional regions.

## RESULTS

### GerG is a novel regulator of *C. difficile* spore germination.

**FIG 1:**
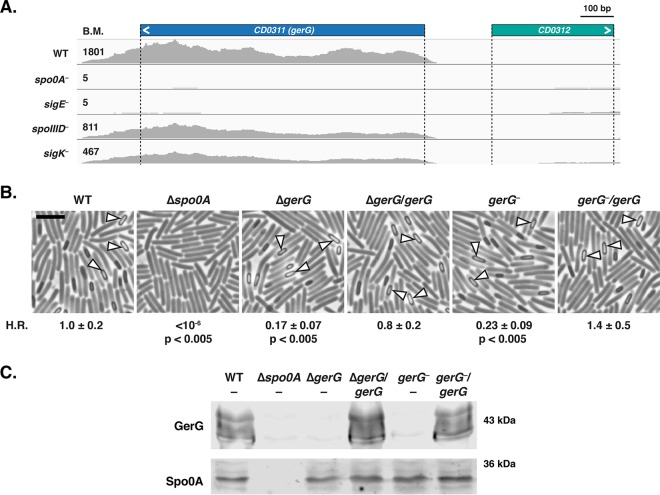
The highly expressed, σ^E^-controlled *gerG* gene contributes to *C. difficile* heat resistance. (A) Representative image of Integrative Genomics Viewer software (75) used to visualize transcript reads of the *gerG* locus from RNA-Seq analyses of sporulating *C. difficile* cultures (30) from wild-type (WT) JIR8094, *spo0A*-negative, *sigE*-negative, *spoIIID*-negative, and *sigK*-negative strains. σ^E^ activates the transcription of *spoIIID*, which encodes a transcriptional regulator that activates *sigK* transcription (53). The angled bracket denotes the direction of transcription. The average base mean (BM) refers to the number of transcripts detected for the respective gene normalized to the length of that gene. The difference in transcript levels detected between wild-type, *spoIIID*-negative, and *sigK*-negative strains did not meet the cutoff of >4-fold change defined in reference 30. (B) Phase-contrast microscopy of sporulating cells of the wild type (630Δ*erm*-p), the Δ*spo0A* mutant, the Δ*gerG* mutant and its complement (Δ*gerG*/*gerG*), and the *gerG*::*ermB* (*gerG*-negative) TargeTron disruption strain and its complement (*gerG*-negative/*gerG*). The Δ*spo0A* mutant cannot initiate sporulation (13), but phase-bright (fore)spores are visible in all other strains (white triangles). H.R. represents the heat resistance of each strain relative to the wild type as determined from three biological replicates. The means and standard deviations shown are based on three biological replicates. Statistical significance relative to the wild type was determined using one-way analysis of variance and Tukey’s test. Bar, 5 µm. (C) Western blot analysis of GerG and the Spo0A loading control (76) in sporulating cells of indicated strains.

In previous work, we used transcriptome sequencing (RNA-Seq) analyses (29, 30) to inform the targeted mutagenesis of *gerS*, as it was one of the most highly induced genes during sporulation and encoded a conserved hypothetical protein (22). Using a similar rationale, we targeted *gerG* for mutagenesis, since it is the 10th most highly expressed, sporulation-induced gene whose function was unknown. Similar to *gerS*, expression of *gerG* is under the control of the mother cell-specific sigma factor E (σ^E^ [30, 31]) (Fig. 1A). Interestingly, GerG appears to be conserved exclusively in *C. difficile*, unlike previously identified germination regulators.

To test whether GerG plays a role in regulating sporulation and/or germination, we constructed TargeTron mutations in *gerG* in the JIR8094 and 630Δ*erm*Δ*pyrE* strain backgrounds as well as a clean deletion of *gerG* in the 630Δ*erm*Δ*pyrE* background (see Fig. S1 in the supplemental material). The latter strain background provides the advantage of allowing mutations to be complemented in single copy on the chromosome at the *pyrE* locus (32), in contrast with the plasmid-based complementation (33) typically used for TargeTron mutants (34) in strains with an intact *pyrE* gene like JIR8094. Both the *gerG* deletion (Fig. 1B) and TargeTron (Fig. S2A) mutants produced phase-bright spores with no obvious defects in either the morphology or frequency of spore formation. However, when functional spore formation was measured using a heat resistance assay (29, 35), 630Δ*erm gerG* mutants exhibited an ~4- to 5-fold defect relative to the wild type (Fig. 1B, *P* < 0.005), and the JIR8094 *gerG* TargeTron mutant exhibited an ~30-fold defect relative to the wild type (Fig. S2A, *P* < 0.05). Western blot analyses confirmed that *gerG* mutants were defective in producing GerG (Fig. 1C and S2B), while single-copy complementation of *gerG* restored GerG to wild-type levels (Fig. 1C). Multiple bands were detected for GerG in both strain backgrounds, suggesting that it is posttranslationally modified and/or unstable during sporulation.

10.1128/mBio.02085-16.2 FIG S1  Construction of *gerG* (*CD0311*) and *sleC* mutants. Download FIG S1, PDF file, 0.7 MB. Copyright © 2017 Donnelly et al. 2017 Donnelly et al. This content is distributed under the terms of the Creative Commons Attribution 4.0 International license.

10.1128/mBio.02085-16.3 FIG S2  A TargeTron insertion in *CD0311* in the JIR8094 strain background leads to a heat resistance defect. Download FIG S2, PDF file, 0.4 MB. Copyright © 2017 Donnelly et al. 2017 Donnelly et al. This content is distributed under the terms of the Creative Commons Attribution 4.0 International license.

**FIG 2:**
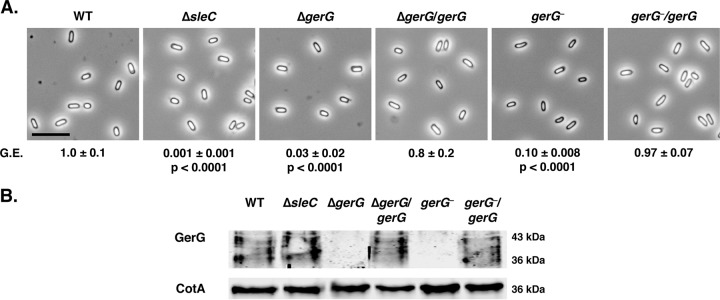
Optimal spore germination depends on GerG. (A) Phase-contrast microscopy of purified spores from the wild type (WT) (630Δ*erm*-p), the Δ*sleC* mutant, the Δ*gerG* mutant and its complement, and the *gerG*::*ermB* (*gerG*-negative) TargeTron disruption strain and its complement. Bar, 5 µm. The germination efficiency (G.E.) of the strains relative to the wild type is shown. The means and standard deviations shown are based on three biological replicates. Statistical significance was determined relative to the wild type using one-way analysis of variance and Tukey’s test on the number of colonies formed by spores when plated on BHIS containing taurocholate. No statistically significant difference in germination efficiency was observed between the Δ*gerG* and *gerG*-negative strains. (B) Western blot analyses of GerG and a CotA loading control in purified spores of the indicated strains.

Since a recent transposon mutagenesis study indicated that insertional mutations in the *gerG* homolog of the epidemic strain R20291 resulted in an ~5-fold decrease in spore purification yields (36), we compared the spore purification yields of the Δ*gerG* mutant relative to those of its complementation strain and the wild type. No statistically significant difference in spore purification was observed for Δ*gerG* spores relative to wild-type 630Δ*erm* (see Table S1 at https://drive.google.com/file/d/0B0M1PLMSo_vDb24yTXNKVFZQaTA/view?usp=sharing). In contrast, Δ*gerG* and *gerG*::*ermB* (*gerG*-negative) spores exhibited 30- and 10-fold decreases in germination efficiency relative to the wild type, respectively, when plated on brain heart infusion-supplemented (BHIS) plates containing taurocholate (TA) (Fig. 2, *P* < 0.0001). JIR8094 *gerG*::*ermB* spores exhibited a 500-fold defect in germination efficiency relative to the wild type (Fig. S3, *P* < 0.0005). JIR8094 *gerG*-minus may have exhibited a more severe spore germination defect than its heat resistance defect because of the high variability in sporulation levels between replicates of our heat resistance assays (Fig. S2). Regardless, these results imply that loss of GerG impairs spore germination rather than spore formation. Importantly, the germination defect of all *gerG* mutant spores could be complemented to wild-type levels (Fig. 2 and S4B), even though GerG was strongly overproduced when *gerG* was expressed from a multicopy plasmid in the JIR8094 *gerG* mutant (Fig. S4B). Interestingly, multiple species of GerG were detected at a lower apparent molecular weight in mature spores (Fig. 2B) than in sporulating cells (Fig. 1C), suggesting that GerG undergoes posttranslational changes during its incorporation into spores.

10.1128/mBio.02085-16.4 FIG S3  GerG is required for optimal spore germination in JIR8094. Download FIG S3, PDF file, 0.4 MB. Copyright © 2017 Donnelly et al. 2017 Donnelly et al. This content is distributed under the terms of the Creative Commons Attribution 4.0 International license.

10.1128/mBio.02085-16.5 FIG S4  Overexpression of *cspBAC* fails to rescue the germination defect of *gerG*-negative spores in JIR8094. Download FIG S4, PDF file, 0.5 MB. Copyright © 2017 Donnelly et al. 2017 Donnelly et al. This content is distributed under the terms of the Creative Commons Attribution 4.0 International license.

In the germination assays shown in Fig. 2 and S3, we used a *sleC* deletion mutant as a negative control (Fig. S1), since *sleC* mutants do not hydrolyze their cortex and thus fail to germinate in at least two strain backgrounds (21, 23, 37). However, in 630Δ*erm*, the Δ*sleC* mutant had only an ~3-log defect in spore germination (Fig. 2; see Table S2 at https://drive.google.com/file/d/0B0M1PLMSo_vDb24yTXNKVFZQaTA/view?usp=sharing), whereas the JIR8094 *sleC*::*ermB* TargeTron mutant (*sleC*-negative) exhibited an ~5-log germination defect (Fig. S3; see Table S2 at https://drive.google.com/file/d/0B0M1PLMSo_vDb24yTXNKVFZQaTA/view?usp=sharing). *sleC* mutant spores outgrew to produce colonies more slowly than wild-type and *gerG* spores when germinated on BHIS plates containing taurocholate in both strain backgrounds (data not shown). For 630Δ*erm*Δ*sleC*, spore outgrowth was not visible until ~16 to 20 h (>4-h delay). If spore germination was assayed earlier than this time point, 630Δ*erm*Δ*sleC* spores appeared to be completely defective in germination. Spore germination did not increase after prolonged incubation (>40 h) for wild-type, *gerG*, or *sleC* mutant spores in both strain backgrounds. Notably, colony formation by *gerG* mutant spores on BHIS-TA plates exhibited kinetics similar to those of the wild type (data not shown). These observations suggest that GerG likely does not affect germinant accessibility, in contrast with the delayed-germination phenotype observed for *Bacillus gerP* mutant spores, which exhibit decreased permeability to germinants (38–40).

While the Δ*sleC* mutant in our hands exhibited an ~3-log defect in spore germination, TargeTron disruption of *sleC* in 630Δ*erm* has been reported to cause an ~4-log germination defect (37). This difference in phenotypes could be due to differences in the nature of the mutations (gene deletion versus disruption) or germination conditions. To distinguish between these possibilities, we reconstructed the *sleC* TargeTron mutation in the 630Δ*erm*Δ*pyrE* strain background (Fig. S1) and restored its *pyrE* locus. The resulting 630Δ*erm sleC*::*ermB* (*sleC*-negative) mutant had an ~3-log germination defect similar to 630Δ*erm*Δ*sleC*, which is ~100-fold less severe than JIR8094 *sleC*::*ermB* (see Table S2 at https://drive.google.com/file/d/0B0M1PLMSo_vDb24yTXNKVFZQaTA/view?usp=sharing). The difference between our results and those of Burns et al. (37) may be due to differences in spore preparation conditions, as has been described previously (41–43). Consistent with this possibility, we observed a >10-fold variation in *sleC* mutant spore germination between spore preparations (see Table S3 at https://drive.google.com/file/d/0B0M1PLMSo_vDb24yTXNKVFZQaTA/view?usp=sharing).

In spite of these differences, the germination defect of all *sleC* mutations could be complemented in both the 630Δ*erm* and JIR8094 strain backgrounds (see Table S2 at https://drive.google.com/file/d/0B0M1PLMSo_vDb24yTXNKVFZQaTA/view?usp=sharing), and artificial germination of 630Δ*erm* Δ*sleC* and Δ*gerG* spores using thioglycolate and lysozyme rescued the ability of these spores to produce colonies to wild-type levels (Fig. S5). Altogether, the results suggest that there are strain-specific differences in the requirement for SleC during spore germination.

10.1128/mBio.02085-16.6 FIG S5  Artificial germination bypasses the need for GerG. Download FIG S5, PDF file, 0.1 MB. Copyright © 2017 Donnelly et al. 2017 Donnelly et al. This content is distributed under the terms of the Creative Commons Attribution 4.0 International license.

### GerG is required for cortex hydrolysis.

**FIG 3:**
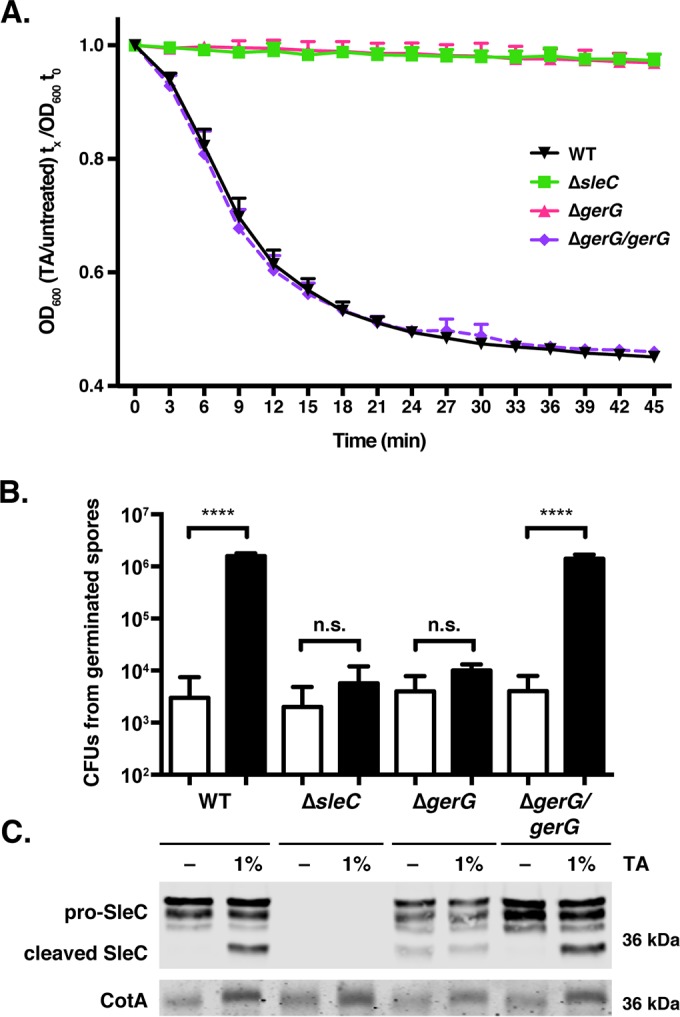
GerG is required for activation of the SleC cortex hydrolase. (A) Change in optical density at 600 nm (OD_600_) of the indicated spores in response to germinant. Δ*sleC* spores were used as a negative control, since SleC is required for cortex hydrolysis (23). OD_600_ measurements were taken every 3 min. Wild-type (WT) and Δ*gerG*/*gerG* spores exhibited statistically significant differences in OD_600_ over time relative to Δ*sleC* and Δ*gerG* spores (*P* < 0.0001); the latter two strains did not exhibit statistically significant differences in OD_600_ over time based on two-way analyses of variance using Tukey’s test. (B) CFU that arose from spores exposed to either water (white bars) or TA germinant (1%, 19 mM; black bars) for 20 min at 37°C and plated on BHIS. Statistical significance relative to the wild type was determined using a one-way analysis of variance and Tukey’s test. n.s., no statistical significance; ****, *P* < 0.0001. (C) Western blot analysis of SleC and CotA (loading control) in samples shown in panel B. −, exposure to water. The pro-SleC zymogen is processed in response to germinant by CspB (21).

Having established that GerG is necessary for optimal spore germination, we sought to identify the stage of germination affected by loss of GerG. We first monitored changes in the optical density at 600 nm (OD_600_) of Δ*gerG* spores relative to the wild type, since the optical density of germinating spores decreases due to cortex hydrolysis and subsequent core expansion (24). When wild-type and Δ*gerG*/*gerG* spores were incubated with 1% taurocholate (19 mM) at room temperature for 45 min, their optical density decreased by ~50% (Fig. 3A); in contrast, the optical density of Δ*gerG* and Δ*sleC* spores did not change appreciably. Since changes in spore optical density in this assay depend on cortex hydrolysis (23), Δ*gerG* spores would appear to be defective in cortex hydrolysis.

Based on these observations, we predicted that the cortex hydrolase SleC fails to undergo proteolytic activation by CspB (21), with the caveat that *gerS*::*ermB* spores can still cleave pro-SleC despite failing to hydrolyze cortex (22). To test this hypothesis, wild-type, Δ*gerG*, and Δ*gerG*/*gerG* spores were exposed to germinant for 20 min at 37°C, and pro-SleC cleavage was monitored by Western blotting (Fig. 3C). Whereas wild-type and Δ*gerG*/*gerG* spores cleaved pro-SleC in response to taurocholate addition, no change in pro-SleC processing was observed in Δ*gerG* spores. Interestingly, low levels of processed SleC were detectable in Δ*gerG* spores even in the absence of germinant, indicating that GerG alters the protease susceptibility of SleC in either sporulating cells or mature spores.

To correlate SleC cleavage with spore germination and outgrowth, we measured the number of spores that had germinated after the 20-min exposure to taurocholate by plating wild-type, Δ*gerG*, and Δ*sleC* spores on medium lacking germinant. An ~1,000-fold increase in CFU was observed when wild-type and Δ*gerG/gerG* spores were transiently exposed to taurocholate germinant and plated on medium lacking germinant relative to untreated spores (Fig. 3B, *P* < 0.0001). In contrast, transient exposure of Δ*gerG* and Δ*sleC* spores to germinant did not significantly increase the number of CFU recovered on medium lacking germinant (Fig. 3B). Taken together, CspB-mediated activation of SleC cortex hydrolase activity does not occur in the absence of GerG.

### GerG controls the levels of Csp germination regulators in spores.

**FIG 4:**
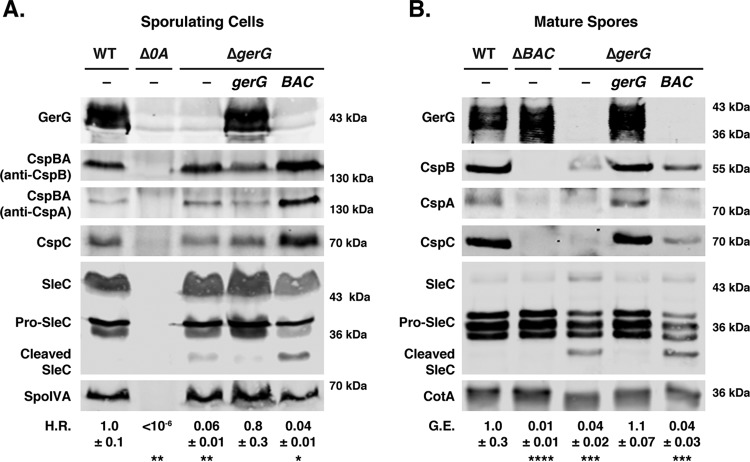
GerG controls Csp levels in mature spores. Western blot analyses of germination regulators in sporulating cells (A) and purified spores (B) from the wild type (WT) (630Δ*erm*-p), the Δ*gerG* mutant, and the Δ*gerG* mutant complemented with either *gerG* (Δ*gerG*/*gerG*) or the *cspBAC* operon (Δ*gerG*/*cspBAC*). The Δ*spo0A* mutant was used as a negative control for sporulating cells, since this strain does not sporulate (13); a Δ*cspBAC* mutant was used as a negative control for analyzing Csp levels in mature spores. CspBA is the primary form detected in sporulating cells, whereas CspB and CspA domains are the primary form detected in mature spores (27). SleC is detected in three major forms: full length (SleC), zymogen (pro-SleC), and proteolytically activated (cleaved SleC [21]). Antibodies against SpoIVA and CotA were used as a loading controls. H.R. represents the heat resistance of each strain relative to the wild type; G.E. refers to the germination efficiency of each strain relative to the wild type. The means and standard deviations shown are based on three biological replicates. Statistical significance relative to the wild type was determined using a one-way analysis of variance and Tukey’s test. ****, *P* < 0.0001; ***, *P* < 0.0005; **, *P* < 0.005; *, *P* < 0.05.

Given that the signaling pathway that leads to CspB-mediated activation of the SleC cortex hydrolase involves CspC sensing germinant (20) and CspA modulating CspC incorporation into spores (27), we tested whether GerG affects the levels of Csp proteins during sporulation. In particular, we analyzed the levels of CspBA and CspC in sporulating cells and CspB, CspA, and CspC in purified spores using immunoblotting. While CspBA and CspC levels were unchanged in sporulating Δ*gerG* cells (Fig. 4A), the levels of CspB, CspA, and CspC were severely reduced in Δ*gerG* spores relative to the wild type (Fig. 4B). Importantly, complementation of Δ*gerG* restored wild-type levels of Csps in spores, indicating that GerG regulates the incorporation of Csp germination regulators into spores and/or their stability in mature spores.

Based on these findings, we wondered whether overproduction of Csp family members might rescue the germination defect of Δ*gerG* spores. To this end, we introduced a second copy of the *cspBA-cspC* operon into the *pyrE* locus of Δ*gerG* (Δ*gerG*/*BAC*). The extra copy of *cspBA-cspC* increased CspBA and CspC levels in sporulating cells above those observed in wild type (Fig. 4). While the levels of CspB and CspC were increased in mature Δ*gerG*/*cspBAC* spores relative to Δ*gerG* spores (Fig. 4), they were still lower than those in the wild type. The increased Csp levels, however, did not affect sporulating cell heat resistance or spore germination efficiency in Δ*gerG*/*cspBAC* spores relative to Δ*gerG* spores.

Since the moderate increase in Csp levels in Δ*gerG*/*cspBA-cspC* spores may have been insufficient to restore germination to wild-type levels, we tested whether plasmid-based overexpression of *cspBAC* might produce enough CspBA and CspC to bypass the need for GerG to incorporate Csp family members into mature spores. We used JIR8094-based mutants in these assays because previous work from our lab showed that expression of *cspBA-cspC* from the multicopy pMTL83151 plasmid overproduced CspBA and CspC in sporulating cells and increased Csp levels in spores (27). Complementation of the JIR8094 *gerG* mutant with the pMTL83151-*cspBAC* plasmid markedly elevated CspBA and CspC levels in sporulating cells (Fig. S4); however, far less CspB, CspA, and CspC were detected in *gerG*-negative/*cspBAC* spores relative to *cspBAC*-negative/*cspBAC* spores (Fig. S4). Despite a marginal increase in Csp protein levels in *gerG*-negative/*cspBAC* spores relative to *gerG*-negative spores carrying empty vector, no change in heat resistance or germination efficiency was observed between these strains (Fig. S4). Taken together, these results highlight the role of GerG modulating the incorporation of Csp proteins into spores.

The *cspBAC* overexpression experiments used *cspBAC* deletion and TargeTron mutants in the 630Δ*erm* and JIR8094 backgrounds, respectively, as negative controls for measuring Csp levels in spores. Similarly to our observations with *sleC* mutants in these two strain backgrounds, the germination phenotypes of these *cspBAC* mutants differed markedly. JIR8094 *cspBAC*-negative spores exhibited a >5-log germination defect relative to wild-type spores, while 630Δ*erm*Δ*cspBAC* spores displayed an ~2-log germination defect (Fig. 4 and S4). These phenotypes support the notion that 630Δ*erm* spores germinate more readily than JIR8094 spores.

### GerG mutant spores are less responsive to germinant.

The observation that Δ*gerG* spores have lower levels of CspC germinant receptor prompted us to investigate whether they would be less sensitive to germinant, as the levels of transmembrane germinant receptors have been shown to control the responsiveness of *Bacillus subtilis* spores to germinant (44, 45). To test this hypothesis, we monitored the germination of individual Δ*gerG* spores in response to increasing concentrations of germinant using optical trapping (46). In this assay, the decrease in optical refractility of germinating spores is monitored on a single-spore basis over time. The decrease in germinating spore optical refractility corresponds with cortex hydrolysis and CaDPA release in *C. difficile* (47), similarly to analyses of bacterial spores from other species (46, 48, 49). Since spore germination dynamics can be measured on a single-spore basis, this assay allows germination heterogeneity to be assessed in contrast with bulk, population-wide measurements such as the optical density assay in Fig. 3.

**FIG 5:**
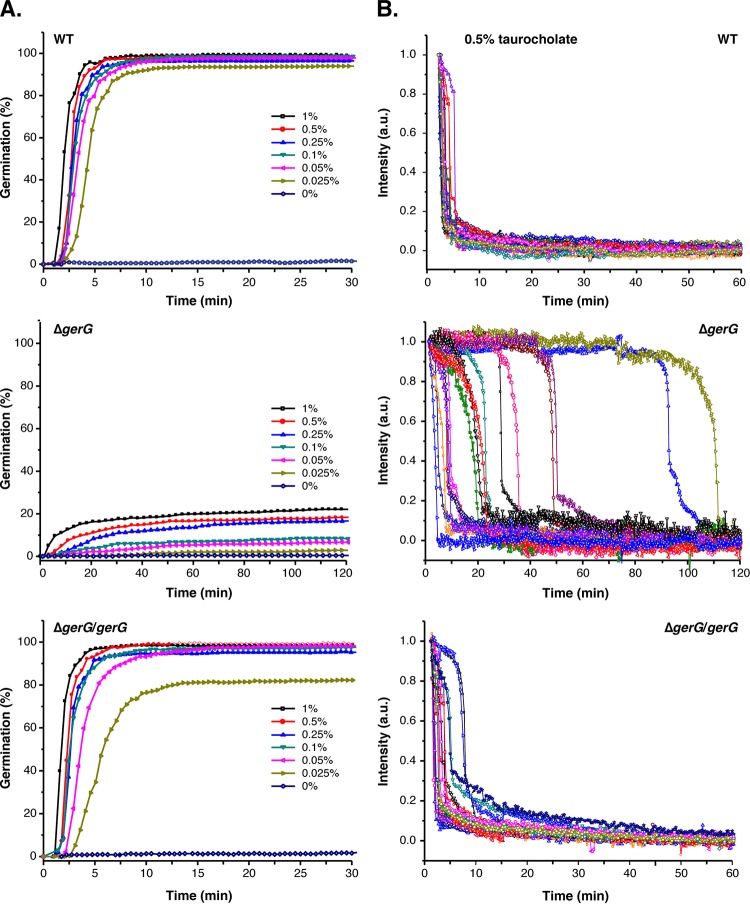
*C. difficile* spores are less responsive to germinant in the absence of GerG. (A) *C. difficile* spore germination in response to increasing concentrations of taurocholate and 15.5 mM glycine at 37°C. Germination responses were measured at the single-spore level by monitoring decreases in phase-contrast image intensity (46). A minimum of 200 spores were monitored for 30 min (wild-type [WT] [630Δ*erm*] and Δ*gerG*/*gerG* strain) or 120 min for the Δ*gerG* strain. (B) Germination responses of individual *C. difficile* spores in the presence of 0.5% taurocholate (9 mM) and 15.5 mM glycine at 37°C. Spores were chosen at random from the subpopulation of germinating spores. The phase-contrast image intensity of a given spore was normalized to 1 based on the respective values at the first time point measured. Image intensities at the end of the experiment were set to zero. a.u., arbitrary units.

When wild-type spores were incubated with the lowest concentration of germinant tested (0.025% taurocholate, 0.5 mM), 95% of the spores completed germination within 10 min at 37°C (Fig. 5; see Table S4 at https://drive.google.com/file/d/0B0M1PLMSo_vDb24yTXNKVFZQaTA/view?usp=sharing). Δ*gerG*/*gerG* complementation spores behaved similarly to the wild type, with 98% of these spores completing germination within 10 min of being exposed to 0.05% taurocholate (0.9 mM). In contrast, only 3% of Δ*gerG* spores completed germination after incubation with 0.025% (0.5 mM) taurocholate for 120 min. While germination was essentially maximal in wild-type and Δ*gerG*/*gerG* spores at 0.25% TA (5 mM), Δ*gerG* spore germination increased in a dose-dependent manner up to the maximum concentration of taurocholate tested (1%, 19 mM; Fig. 5A).

Spore germination was also more heterogeneous in Δ*gerG* spores than in wild-type and Δ*gerG*/*gerG* spores (Fig. 5B). When the germination dynamics of 15 individual Δ*gerG* spores were analyzed, germination was observed between 10 and 120 min of germinant addition. These 15 spores were chosen at random from the subset (<20%) of Δ*gerG* spores that germinated after exposure to 0.5% taurocholate (9 mM) for 120 min. In contrast, all the wild-type and Δ*gerG*/*gerG* spores analyzed in this manner uniformly initiated and completed germination within 10 min of germinant addition. Although Δ*gerG* spores exhibited greater variability in the time to initiate germination than did the wild type, Δ*gerG* spores completed germination with similar kinetics as the wild-type spores as evidenced by the kinetics of the sharp decrease in optical refractility. Since this decrease corresponds to cortex hydrolysis and CaDPA release (47, 50), GerG would appear to control the initiation of germination rather than the kinetics of these germination events. This finding is consistent with the observation that the Δ*gerG* spores form colonies at a similar rate as the wild type when germinated on BHIS plates containing taurocholate, whereas Δ*sleC* spores form colonies with a >4-h delay (data not shown).

To assess whether the levels of germination measured in the optical trapping assay corresponded to spore outgrowth, we determined the colony-forming capacity of wild-type, Δ*gerG*, and Δ*gerG*/*gerG* spores exposed to increasing concentrations of taurocholate. Spores were exposed to taurocholate for 20 min at 37°C, and the number of spores that initiated germination was enumerated by plating on BHIS plates lacking germinant. Almost all of the wild-type and Δ*gerG*/*gerG* spores initiated germination upon being exposed to 0.5% taurocholate (9 mM) for 20 min (Fig. S6), since similar numbers of CFU were produced when taurocholate-treated spores were plated on BHIS plates lacking germinant as when untreated spores were plated on BHIS plates containing taurocholate germinant (dashed line). In contrast, Δ*gerG* spores needed to be exposed to 10-fold-higher levels of taurocholate (>5%, 86 mM) in order to achieve maximal Δ*gerG* spore germination, i.e., CFU comparable to those observed when untreated Δ*gerG* spores were plated on BHIS containing taurocholate (dashed line). These results are largely consistent with the single-spore germination analyses, although the germination defect of Δ*gerG* spores relative to wild-type spores was slightly more severe in the spore outgrowth assay (15-fold decrease at 0.5% TA, *P* < 0.0001; Fig. S6A) than the optical trapping assay (~5-fold decrease at 0.5% TA; see Table S4 at https://drive.google.com/file/d/0B0M1PLMSo_vDb24yTXNKVFZQaTA/view?usp=sharing). Interestingly, Western blot analyses of Δ*gerG* spores exposed to supraphysiological concentrations of taurocholate (8, 51) did not show increased SleC cleavage even though greater levels of Δ*gerG* spores germinated and outgrew to form colonies on BHIS plates (Fig. S6B).

10.1128/mBio.02085-16.7 FIG S6  Responsiveness of *gerG* mutant spores to germinant. Download FIG S6, PDF file, 0.5 MB. Copyright © 2017 Donnelly et al. 2017 Donnelly et al. This content is distributed under the terms of the Creative Commons Attribution 4.0 International license.

### Asparagine-rich internal repeat regions are dispensable for GerG function.

**FIG 6:**
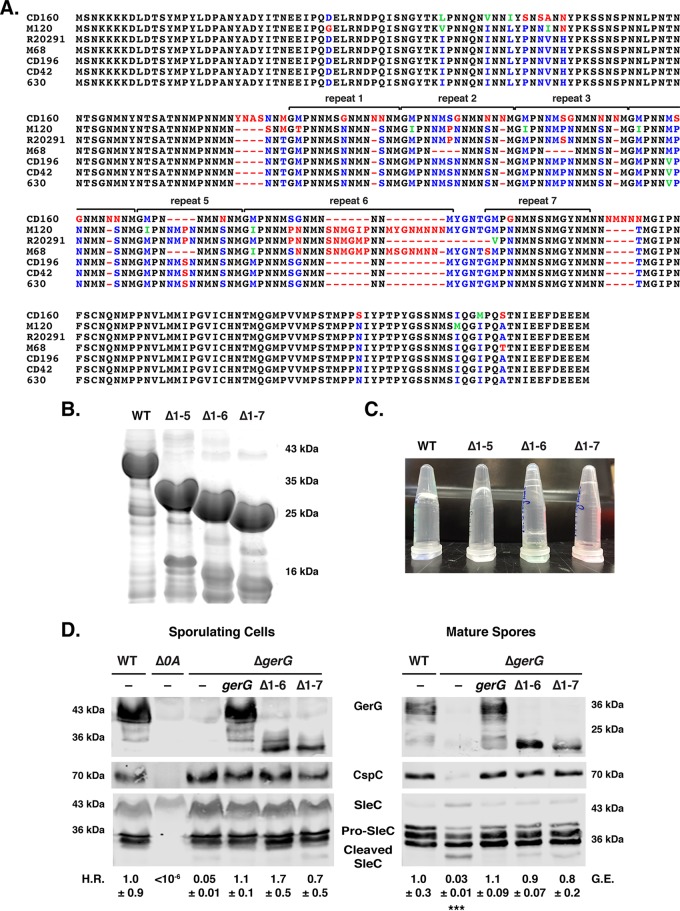
The central repeat region of GerG is required for gel formation but dispensable for spore germination. (A) ClustalW alignment of GerG from selected *C. difficile* strains. Completely conserved residues are colored black, conserved identical residues are in blue, conserved similar residues are in green, and non-conserved residues are in red. (B) Coomassie blue staining of recombinant, affinity-purified, His-tagged GerG variants. The repeat sequences shown in panel A that were deleted are shown. WT, wild type. (C) Gel formation of GerG variants shown in panel B as determined by inverting the purified protein preparations. (D) Western blot analyses of GerG, CspC, and SleC in sporulating cells and purified spores of the wild type, the Δ*spo0A* mutant, the Δ*gerG* mutant, and the Δ*gerG* strain complemented with either wild-type *gerG* or *gerG* encoding deletions of repeats 1 to 6 (Δ1–6) or 1 to 7 (Δ1–7). H.R. represents the heat resistance of each strain relative to the wild type; G.E. refers to the germination efficiency of each strain relative to the wild type. The means and standard deviations shown are based on three biological replicates. Statistical significance relative to the wild type was determined using a one-way analysis of variance and Tukey’s test. ***, *P* < 0.0005. No statistically significant difference was observed between the strains used in the heat resistance assay due to high variance in sporulation levels. No statistically significant difference in germination efficiency was observed between Δ1–6 and Δ1–7 relative to the wild type.

Having demonstrated that GerG modulates Csp incorporation into spores and thus appears to affect their responsiveness to germinant, we next sought to identify functional regions within this previously uncharacterized protein. GerG has a highly unusual amino acid composition: ~30% of its residues are asparagines, 15% are methionines, 11% are prolines, 8% are serines, and 6% are glycines. Most of these residues are concentrated in a central repeat region, which consists of GMPNNMSNNMNSNM repeats that vary slightly in composition and number among *C. difficile* strains (Fig. 6A).

Interestingly, when recombinant His-tagged GerG was purified (Fig. 6B) to generate antibodies, the protein spontaneously formed a gel overnight (Fig. 6C). GerG gel formation appeared to occur in a concentration-dependent manner (see Table S5 at https://drive.google.com/file/d/0B0M1PLMSo_vDb24yTXNKVFZQaTA/view?usp=sharing), with concentrations of ~200 µM being sufficient to permit gel formation. However, since concentrating GerG-His_6_ did not necessarily lead to gel formation (data not shown), this phenomenon may depend on high concentrations of GerG-His_6_ being immobilized in close proximity on nickel affinity beads. Gel formation was observed at 4°C, 25°C, and 37°C, although it occurred more slowly at higher temperatures (data not shown).

Since asparagine-rich proteins with low-complexity regions (LCRs) are prone to aggregation (52), we tested whether deleting the asparagine-rich repeat sequences would prevent gel formation. Strain 630Δ*erm* contains seven internal repeat regions (Fig. 6A), and deletion of the first five did not abolish gel formation (Fig. 6C; see Table S5 at https://drive.google.com/file/d/0B0M1PLMSo_vDb24yTXNKVFZQaTA/view?usp=sharing). Deletion of six or all of its repeat regions, however, prevented gel formation at >600 µM protein concentrations (Fig. 6C; see Table S5 at https://drive.google.com/file/d/0B0M1PLMSo_vDb24yTXNKVFZQaTA/view?usp=sharing).

To assess whether the repeat regions were important for GerG to regulate spore germination, we complemented Δ*gerG* spores with constructs lacking six (Δ1–6) or all (Δ1–7) of the repeat sequences. Remarkably, the Δ1–6 and Δ1–7 constructs fully complemented the heat resistance and germination defects of the parental Δ*gerG* strain (Fig. 6C). CspC levels in Δ*gerG*/Δ1–6 and Δ*gerG*/Δ1–7 strains were restored to wild-type levels by the GerG truncations, even though the truncations themselves reduced the overall levels of GerG produced (Fig. 6C) as detected by an antibody raised against recombinant GerG(Δ1–7). Taken together, our results indicate that the repeat region is dispensable for GerG function in *C. difficile*, even though this region mediates GerG gel formation *in vitro*.

## DISCUSSION

The regulators controlling spore germination in *C. difficile* differ markedly from those identified in *Bacillus* and *Clostridium* spp. to date. In particular, the CspC germinant receptor, the CspA pseudoprotease domain, and the GerS lipoprotein appear to be unique to the *Peptostreptococcaceae* family. In this report, we identified GerG as a *C. difficile-*specific factor that is required for optimal *C. difficile* spore germination, further underscoring the uniqueness of the *C. difficile* germination pathway. We find that the levels of key players in the *C. difficile* germinant signaling pathway, CspA, CspB, and CspC, are dramatically reduced in *gerG* mutant spores (Fig. 4 and S4) and that this decrease correlates with reduced responsiveness to germinant (Fig. 5 and S6).

An obvious question that arises from our study is how GerG regulates Csp levels in mature spores. While Csp family members are produced in the mother cell (29, 31, 53), they almost certainly function in the intermembrane space between the mother cell and forespore based on the following observations. During germination, the CspB protease must access its target, SleC, which is localized in the cortex (54); thus, CspB is presumably also localized to the cortex. Additionally, CspC activates CspB in response to germinant (20) through a proposed direct binding event (27, 55), making CspC likely to also be localized to the cortex region. Finally, CspA regulates CspC incorporation into spores (27) via a putative interaction, suggesting a cortex region localization for CspA as well. In support of these predicted protein-protein interactions, it has previously been shown that subtilisin-like serine proteases can form higher-order structures (56). Taken together, we predict that the ability of Csp proteins to control *C. difficile* spore germination depends on their transport across the mother cell-derived outer forespore membrane. Based on these assumptions, we speculate that GerG facilitates the transport of Csp proteins into the cortex region. Consistent with this hypothesis, overproduction of the Csps fails to substantially increase their levels in mature spores (Fig. S4). Since the Csp proteins that were incorporated into these spores (*gerG*::*ermB*/*cspBAC* spores; Fig. S4) remained unable to function properly, we speculate that they became spore associated as the coat assembled around the forespore. Unfortunately, this hypothesis cannot be tested in the absence of fractionation methods that can specifically isolate coat proteins without also extracting proteins in the intermembrane space (e.g., cortex region) (22, 57).

While these issues remain to be resolved, our results suggest that GerG acts upstream of CspA, since loss of GerG reduces levels of all three Csp germination regulators in spores (Fig. 4), whereas loss of CspA reduces levels of CspC but not CspB (27). Another clue to GerG’s function may be the low levels of pro-SleC processing observed in dormant *gerG* mutant spores (Fig. 3 and 4), which suggest that GerG prevents improper associations between CspB and SleC in sporulating cells and/or mature spores. Investigating the putative binding partners of GerG should provide insight into its precise mechanism of action.

Additional insight into GerG function will likely come from delineating its functional domains. Our results indicate that the central, asparagine-rich repeat region of GerG is dispensable for spore germination under the conditions tested (Fig. 6C), even though it regulates gel formation *in vitro* (Fig. 6B). Interestingly, this region varies in the number of repeats (typically between 6 and 7) in expanded analyses of *C. difficile* GerG homologs (see Fig. S7 in the supplemental material). These observations suggest that there may be selective pressure to maintain these repeats, especially since DNA replication slippage could alter the number of repeats (see Materials and Methods). By analogy, a DNA-based mechanism for expanding regions encoding asparagine-rich sequences has been observed in the AT-rich genome of the parasite *Plasmodium falciparum*, and these regions have been shown to be under positive selection (58).

10.1128/mBio.02085-16.8 FIG S7  ClustalW alignment of expanded GerG homologs. Download FIG S7, PDF file, 0.1 MB. Copyright © 2017 Donnelly et al. 2017 Donnelly et al. This content is distributed under the terms of the Creative Commons Attribution 4.0 International license.

The repeat region is predicted to be part of a larger region predicted to be disordered based on analyses using Predictors of Natural Disorder (PONDR [59]) (Fig. S8). Naturally disordered proteins often contain regions of low complexity, i.e., regions with little diversity in their amino acid composition, similar to GerG. Since low-complexity region (LCR) proteins can become structured upon interacting with binding partners and have been observed to have more binding partners in protein-protein interaction networks (60, 61), it is tempting to speculate that GerG may directly bind the Csp germination regulators in sporulating cells.

10.1128/mBio.02085-16.9 FIG S8  PONDR plot analysis of GerG. Download FIG S8, PDF file, 0.3 MB. Copyright © 2017 Donnelly et al. 2017 Donnelly et al. This content is distributed under the terms of the Creative Commons Attribution 4.0 International license.

Interestingly, asparagine-rich proteins have also been associated with prion formation (52, 62). Bioinformatic analysis of GerG using the PLAAC (Prion-Like Amino Acid Composition [63]) application predicts that the majority of GerG constitutes a prion-forming domain (PrD, amino acids [aa] 43 to 270; Fig. S9). The capacity of GerG to self-aggregate (Fig. 6C) in a time- and concentration-dependent manner (see Table S5 at https://drive.google.com/file/d/0B0M1PLMSo_vDb24yTXNKVFZQaTA/view?usp=sharing) is consistent with a propensity to form amyloid-like aggregates (62). It will be interesting to test whether GerG has prionogenic properties and the functional relevance of such a result.

10.1128/mBio.02085-16.10 FIG S9  PLAAC analysis of GerG. Download FIG S9, PDF file, 0.4 MB. Copyright © 2017 Donnelly et al. 2017 Donnelly et al. This content is distributed under the terms of the Creative Commons Attribution 4.0 International license.

Another question raised by our study is why Δ*gerG* spore germination is so heterogeneous (Fig. 5B). Germination heterogeneity may be related to the amount of Csps in a given Δ*gerG* spore, since the levels of germinant receptor have been shown in other systems to control the responsiveness of spores to germinant (44, 45). If Csp levels are the primary driver of spore germination heterogeneity, one could imagine that nongerminating *gerG* spores either are devoid of Csps (i.e., a bimodal distribution) or carry insufficient levels of Csps to meet the threshold level needed to initiate germination (i.e., normal distribution). Given that Δ*gerG* spore germination is dose dependent (Fig. 5 and S6) and that *gerG* mutant spores do not exhibit delayed germination, our results are more consistent with the latter possibility. We are currently developing fluorescent protein fusions to Csp germination regulators to test this hypothesis.

The reduced responsiveness of Δ*gerG* spores to germinant also raises the possibility that GerG could affect the ability of *C. difficile* to initiate disease. At the lowest concentration of germinant tested (0.025%, 0.5 mM), only 3% of Δ*gerG* spores germinated compared to 95% of wild-type spores after a 120-min and a 60-min exposure to germinant, respectively (see Table S3 at https://drive.google.com/file/d/0B0M1PLMSo_vDb24yTXNKVFZQaTA/view?usp=sharing). This concentration of taurocholate is equivalent to the concentrations measured in the ileum of mice prior to antibiotic treatment (8), the ileum being a location that supports *C. difficile* spore germination in *ex vivo* studies (10). It is also similar to the levels measured in the feces of patients suffering from recurrent *C. difficile* infection (0.55 mM [51]). Thus, the infectious dose of a *gerG* mutant could be diminished under conditions favorable to *C. difficile* infection and ultimately reduce disease severity and recurrence. Reduced responsiveness to germinant has been correlated with decreased recurrence in some studies (64–66), while others have linked it to greater disease severity, with the notion that spores do not prematurely germinate before they have reached the colon (67). Since the clinical isolates used in these studies are genetically diverse, comparing the infectious dose and virulence of wild-type and *gerG* mutant spores would allow this question to be resolved.

If *gerG* mutant spores exhibit virulence defects in animal models of infection, the uniqueness of GerG to *C. difficile* could be exploited for developing therapies that prevent *C. difficile* spore germination without disrupting the normal microbiota. Given that a healthy microbiome confers significant colonization resistance against *C. difficile* infection (7, 9, 12, 68), therapies that target GerG could be more effective at preventing disease recurrence. Testing this possibility would be greatly aided by further study into the functional significance and mechanism of action of this unique protein.

## MATERIALS AND METHODS

### Bacterial strains and growth conditions.

*C. difficile* strains are listed in Table S6 at https://drive.google.com/file/d/0B0M1PLMSo_vDb24yTXNKVFZQaTA/view?usp=sharing and derive from two different parent strains, JIR8094 (630E) and 630Δ*erm*Δ*pyrE*, which are erythromycin-sensitive derivatives of the sequenced clinical isolate 630 (69). 630Δ*erm*Δ*pyrE* is a derivative of 630Δ*erm* that carries a deletion in the gene *pyrE*, which encodes an orotate phosphoribosyltransferase required for uracil prototrophy (32). Despite their shared lineages, 630Δ*erm* and JIR8094 exhibit marked phenotypic differences (70). *C. difficile* strains were typically grown on solid BHIS medium (71) supplemented with taurocholate (TA; 0.1% [wt/vol], 1.9 mM), thiamphenicol (5 to 15 µg/ml), kanamycin (50 µg/ml), cefoxitin (8 µg/ml), FeSO_4_ (50 µM), and/or erythromycin (10 µg/ml) as indicated. During allele-coupled exchange (32), *C. difficile* defined minimal medium (CDMM) was used as previously described (72) supplemented with 5-fluoroorotic acid (5-FOA) at 2 mg/ml and uracil at 5 µg/ml as needed. Cultures were grown at 37°C, under anaerobic conditions using a gas mixture containing 85% N_2_, 5% CO_2_, and 10% H_2_. HB101/pRK24 *Escherichia coli* strains were used for conjugations, and BL21(DE3) strains were used for protein production. *E. coli* strains (see Table S1 at https://drive.google.com/file/d/0B0M1PLMSo_vDb24yTXNKVFZQaTA/view?usp=sharing) were routinely grown at 37°C with shaking at 225 rpm in Luria-Bertani broth (LB). Medium was supplemented with chloramphenicol (20 µg/ml), ampicillin (50 µg/ml), or kanamycin (30 µg/ml) as indicated.

Details of strain construction, antibody production, Western blot analyses, and artificial germination are provided in Text S1 in the supplemental material.

10.1128/mBio.02085-16.1 TEXT S1  Supplemental materials and methods and references. Download TEXT S1, PDF file, 0.1 MB. Copyright © 2017 Donnelly et al. 2017 Donnelly et al. This content is distributed under the terms of the Creative Commons Attribution 4.0 International license.

### Sporulation.

*C. difficile* strains were grown from glycerol stocks on BHIS plates supplemented with TA (0.1% [wt/vol], 1.9 mM). Colonies that arose were then used to inoculate 70:30 agar plates (35, 73, 74) for ~24 h. For JIR8094 strains carrying the pMTL83151 vector, 5 µg/ml thiamphenicol was added to these plates to maintain the plasmid. Sporulating cells were harvested into phosphate-buffered saline (PBS) and washed, and sporulation was assessed by phase-contrast microscopy. The remaining sample was processed as needed.

### Heat resistance assay on sporulating cells.

*C. difficile* strains were induced to sporulate as described above for 24 h, and functional (heat-resistant) spore formation was measured as described in reference 35. Heat resistance efficiencies were determined based on the ratio of heat-resistant cells to total cells for a given strain relative to the ratio determined for the wild type. Results are based on the average ratio obtained from a minimum of three biological replicates.

### Spore purification.

After sporulation was induced on 70:30 plates for 2 to 3 days, spores were purified as previously described (22). Briefly, spores were washed ~5 times in ice-cold water, incubated overnight in water, DNase treated, purified on a HistoDenz gradient (71), and washed with water. Spore purity was evaluated using phase-contrast microscopy, and spores were stored in water at 4°C.

### Germination assay.

Approximately 1 × 10^7^ spores (0.35 OD_600_ units) were resuspended in 100 µl of water, and 1/10 of this mixture was serially diluted in PBS. Ten-microliter dilutions were plated onto prereduced BHIS-TA, and colonies arising from germinated spores were enumerated at ~22 h. For *sleC* mutant spore germination, CFU were monitored for up to 48 h. Germination efficiency represents the average number of CFU produced when spores of a given strain were plated on BHIS-TA relative to the number produced by wild-type spores. At least three biological replicates were performed using at least two independent spore preparations. The rest of the sample was processed for Western blot analyses.

### SleC cleavage analysis.

SleC cleavage was induced as previously described (22) with 1% taurocholate (19 mM) for 20 min at 37°C.

### Optical density analysis of spore germination.

Germination was induced as previously described (24). Briefly, spores in BHIS were exposed to 1% taurocholate (19 mM), and the OD_600_ was measured every 3 min for 45 min. The ratio of the OD_600_ measured for the TA-treated sample to that of the untreated sample for a given time point was calculated and then divided by the ratio determined at 0 min.

### Germinant titration analyses.

Responsiveness of spores to low taurocholate concentrations was measured by resuspending ~4 × 10^7^ spores (~1.4 OD_600_ units) in 160 µl of water. Two hundred microliters of BHIS was added to each spore suspension, and 90-µl aliquots of this suspension were added to 10 µl of either water, 1% TA, or 5% TA (to give a final concentration of 0.025% TA, 0.1% TA, or 0.5% TA, respectively). To determine responsiveness of spores to high taurocholate concentrations, ~5 × 10^7^ spores (1.7 OD_600_ units) were resuspended in 280 µl of BHIS, and 50-µl aliquots of this suspension were added to 50 µl of 2%, 5%, 10%, or 20% TA (to give final concentrations of 1%, 2.5%, 5%, and 10% TA, respectively). The samples were incubated for 20 min at 37°C, and a 10 -µl aliquot was removed for 10-fold serial dilutions into PBS. Ten-microliter dilutions were plated onto BHIS to determine the number of spores that had initiated germination and on BHIS-TA plates to determine the maximum level of spore germination.

### Monitoring single-spore germination by phase-contrast microscopy.

The germination of multiple individual spores was monitored by phase-contrast microscopy as described previously (46). In brief, spores were spread on the surface of a coverslip, and the coverslips were sealed and mounted to a microscope sample holder kept at a constant temperature. Preheated germinant solution was added to the spores on the coverslips, and phase-contrast images of spores were recorded at a rate of 1 frame per 15 s for 60 to 120 min by a digital charge-coupled device camera (16 bits; 1,600 by 1,200 pixels). The averaged pixel intensity of an area of 20 by 20 pixels covering each individual spore was calculated, and the image intensity of each individual spore was plotted as a function of the incubation time with a resolution of 15 s. The initial image intensity at the first time of measurement, *T*0, was normalized to 1, and the intensity at the end of the measurement period was normally set at 0. Invariably, the image intensity was constant for >10 min at the end of measurements. The degree of germination of spore populations was measured by simultaneously monitoring the germination of >200 individual spores by phase-contrast microscopy.

### Assessment of GerG-His_6_ gel formation.

GerG-His_6_ variants were purified on Ni^2+^ affinity resin as previously described from 1 liter of 2YT culture (21). Briefly, culture pellets were resuspended in 25 ml lysis buffer (500 mM NaCl, 50 mM Tris-HCl, pH 7.5, 15 mM imidazole, 10% [vol/vol] glycerol) and flash frozen in liquid nitrogen. The sample was lysed by sonication, and the lysate was cleared by centrifugation prior to incubation with 1.0 ml nickel-nitrilotriacetic acid (Ni-NTA) agarose beads (5 Prime) for 2 to 3 h. After the resin was washed with lysis buffer, the His_6_-tagged proteins were eluted into 325 µl of high-imidazole buffer (500 mM NaCl, 50 mM Tris, pH 7.5, 175 mM imidazole, 10% glycerol) and nutated for 5 to 10 min. Samples were pelleted at low speed, and the supernatant was removed without disturbing the pelleted resin. This procedure was repeated four times, and the concentration of protein in the supernatants was measured using a NanoDrop 2000 spectrophotometer (Thermo Scientific); the high-imidazole buffer was used as the blank. The samples were incubated at 4°C and periodically inverted to assess gel formation. Samples were scored as forming a gel if the sample remained at the bottom of the tube when inverted and shaken. The high-imidazole buffer was stored as a stock solution at 4°C for >1 year; no gel formation was observed in the buffer during this time. Gel formation of the Ger-His_6_ variants was assessed in at least two independent protein purifications.
