# Supplementary material for: A Clostridium difficile-Specific, Gel-Forming Protein Required for Optimal Spore Germination
Source: mBio. 2017 Jan 17;8(1):e02085-16. doi: 10.1128/mBio.02085-16 (PMC5241399; doi:10.1128/mBio.02085-16)
Supplement: FIG S1 [file mbo002173148sf1.pdf]

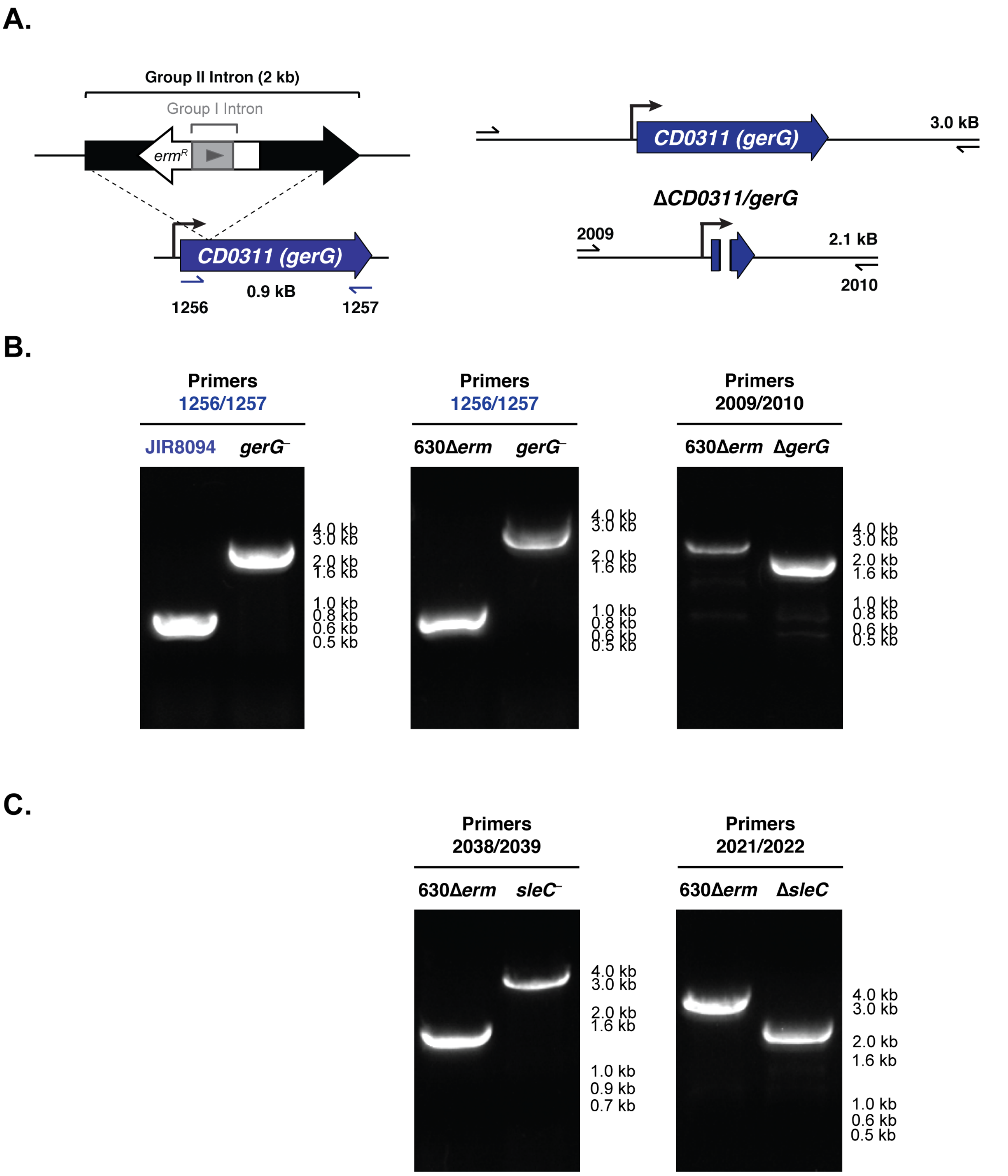

FIG S1. Construction of *gerG* (*CD0311*) and *sleC* mutants. (A) Schematic of the Targetron-based disruption of *gerG* in JIR8094 and 630Δ*erm*Δ*pyrE* (*gerG*::*ermB*) and deletion of *gerG*

(Δ*gerG*) in 630Δ*erm*Δ*pyrE*. (B) Colony PCR of *sleC*::*ermB* and Δ*sleC* constructed in 630Δ*erm*Δ*pyrE*. The group II intron is ~2 kB.
