# Supplementary material for: A Clostridium difficile-Specific, Gel-Forming Protein Required for Optimal Spore Germination
Source: mBio. 2017 Jan 17;8(1):e02085-16. doi: 10.1128/mBio.02085-16 (PMC5241399; doi:10.1128/mBio.02085-16)
Supplement: FIG S2 [file mbo002173148sf2.pdf]

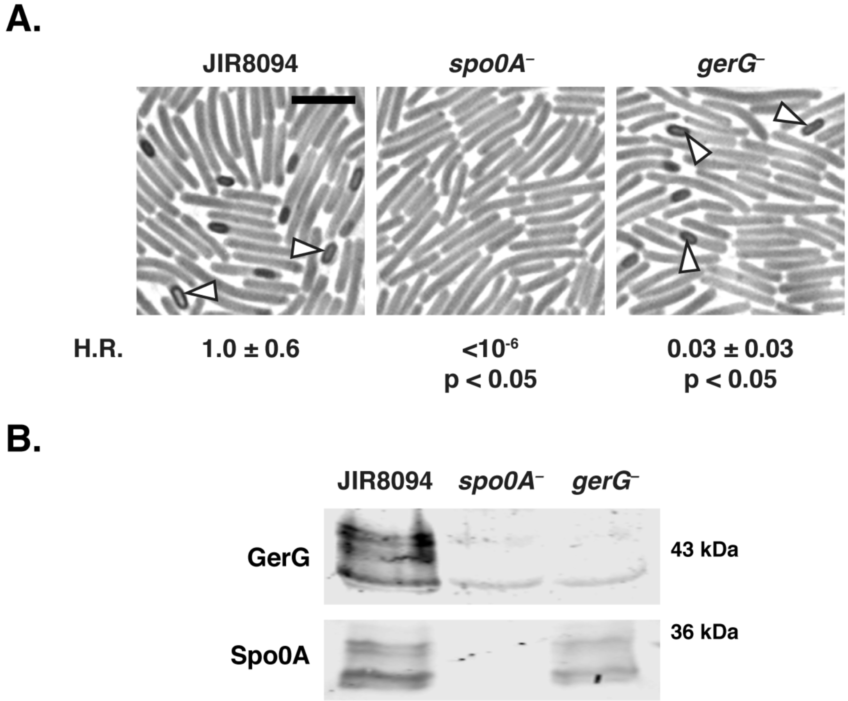

FIG S2. A targetron insertion in *CD0311* in the JIR8094 strain background leads to a heat resistance defect. (A) Phase contrast microscopy of sporulating cells of wild type JIR8094, *spo0A*::*ermB* (*spo0A*$^{-}$), and *gerG*::*ermB* (*gerG*$^{-}$). *spo0A*$^{-}$ cannot initiate sporulation (14). Phase-bright spores are visible in both wild type and *gerG*$^{-}$ (white triangles). H.R. represents the average heat-resistance of each strain relative to wild type as determined from three biological replicates. The standard deviation is shown. Statistical significance relative to wild type was determined using one-way ANOVA and Tukey's test. Scale bars represent 5 μm. (B) Western blot analysis of GerG and the Spo0A loading control (11) in sporulating cells of the indicated strains.
