# Supplementary material for: A Clostridium difficile-Specific, Gel-Forming Protein Required for Optimal Spore Germination
Source: mBio. 2017 Jan 17;8(1):e02085-16. doi: 10.1128/mBio.02085-16 (PMC5241399; doi:10.1128/mBio.02085-16)
Supplement: FIG S3 [file mbo002173148sf3.pdf]

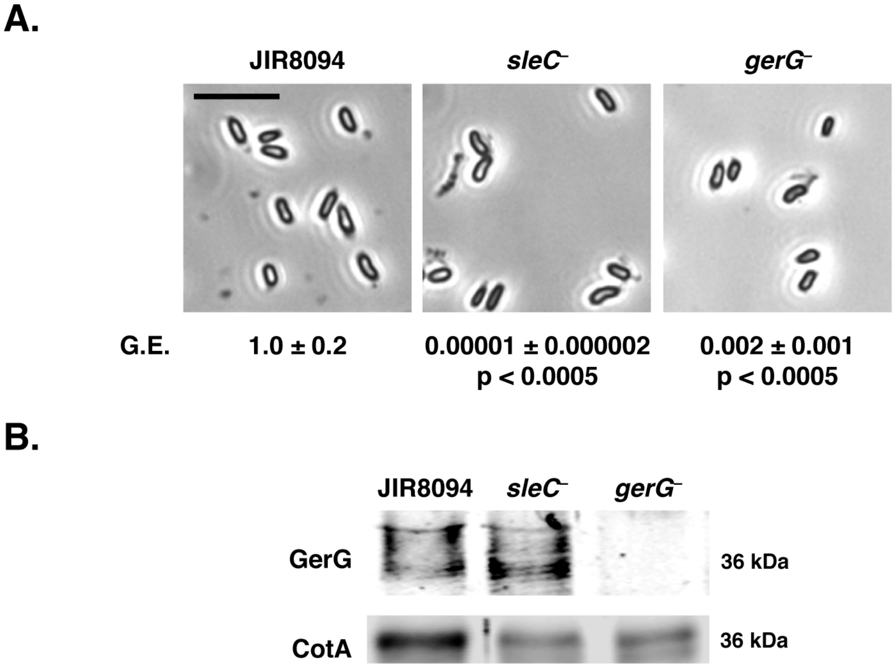

FIG S3. GerG is required for optimal spore germination in JIR8094.

(A) Phase contrast microscopy of wildtype JIR8094, *sleC*⁻, and *gerG*⁻ spores. Scale bars represent 5 µm. The germination efficiency of the strains relative to wild type is shown. Data represents the average and standard deviation of three biological replicates. Statistical significance relative to wild type was determined using one-way ANOVA and Tukey's test. (B) Western blot analyses of GerG and the CotA loading control in purified spores of the indicated strains.
