# Supplementary material for: A Clostridium difficile-Specific, Gel-Forming Protein Required for Optimal Spore Germination
Source: mBio. 2017 Jan 17;8(1):e02085-16. doi: 10.1128/mBio.02085-16 (PMC5241399; doi:10.1128/mBio.02085-16)
Supplement: FIG S4 [file mbo002173148sf4.pdf]

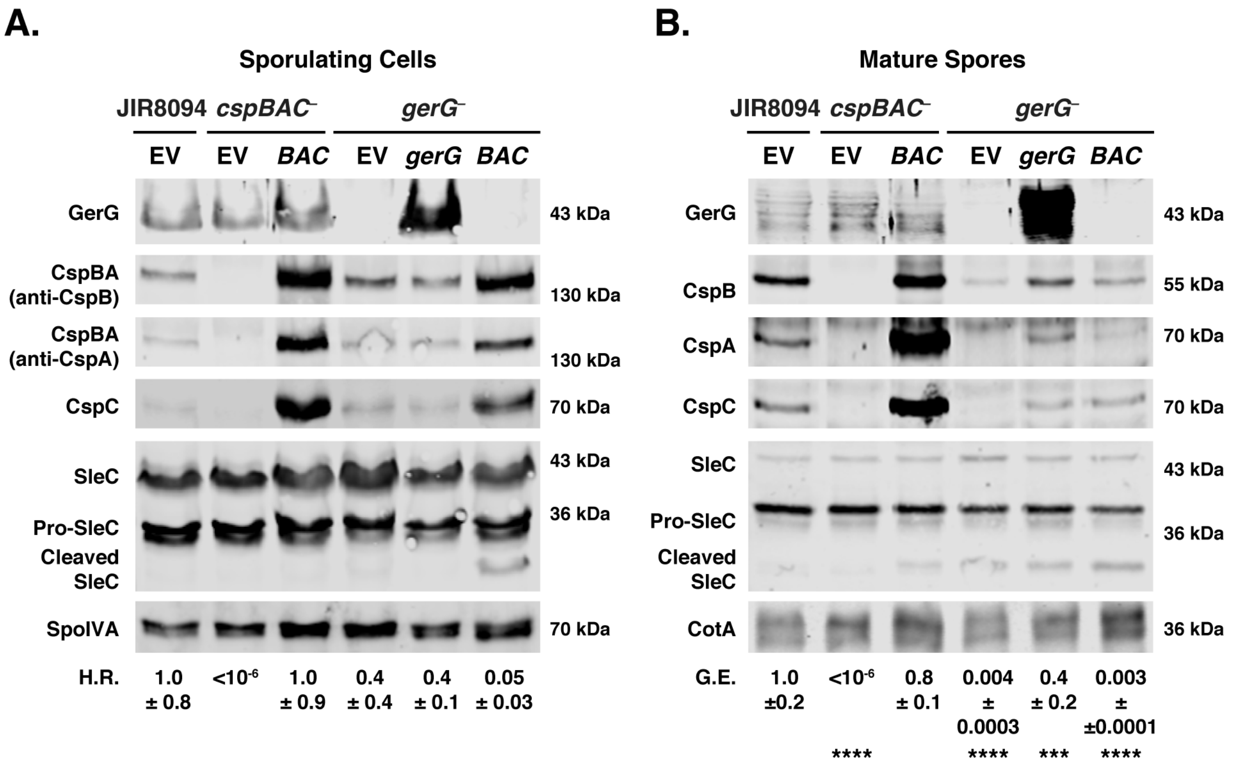

FIG S4. Over-expression of *cspBAC* fails to rescue the germination defect of *gerG*⁻ spores in JIR8094. Western blot analyses of germination regulators in (A) sporulating cells and (B) purified spores from wildtype JIR8094, *cspBAC*⁻, and *gerG*⁻ carrying empty pMTL83151 vector (EV). *cspBAC*⁻ was complemented with *cspBAC* expressed from the pMTL83151 multicopy plasmid, and *gerG*⁻ was complemented with either *gerG* or *cspBAC* expressed from pMTL83151. CspBA is the primary form detected in sporulating cells, whereas CspB and CspA domains are the primary form detected in mature spores (12). SleC is detected in three major forms: full-length (SleC), zymogen (pro-SleC), and proteolytically activated (cleaved SleC, (9)). Antibodies against SpoIVA and CotA were used as loading controls for sporulating and mature spores, respectively. H.R. represents the average heat-resistance of each strain relative to wild type; G.E. refers to average germination efficiency of each strain relative to wild type. The mean and standard deviations shown are based on three biological replicates. Statistical significance

relative to wild type was determined using one-way ANOVA and Tukey's test. **** $p < 0.0001$ and *** $p < 0.0005$. No statistically significant difference was observed between the strains used in the heat resistance assay due to high variance in sporulation levels.
