# Supplementary material for: A Clostridium difficile-Specific, Gel-Forming Protein Required for Optimal Spore Germination
Source: mBio. 2017 Jan 17;8(1):e02085-16. doi: 10.1128/mBio.02085-16 (PMC5241399; doi:10.1128/mBio.02085-16)
Supplement: FIG S5 [file mbo002173148sf5.pdf]

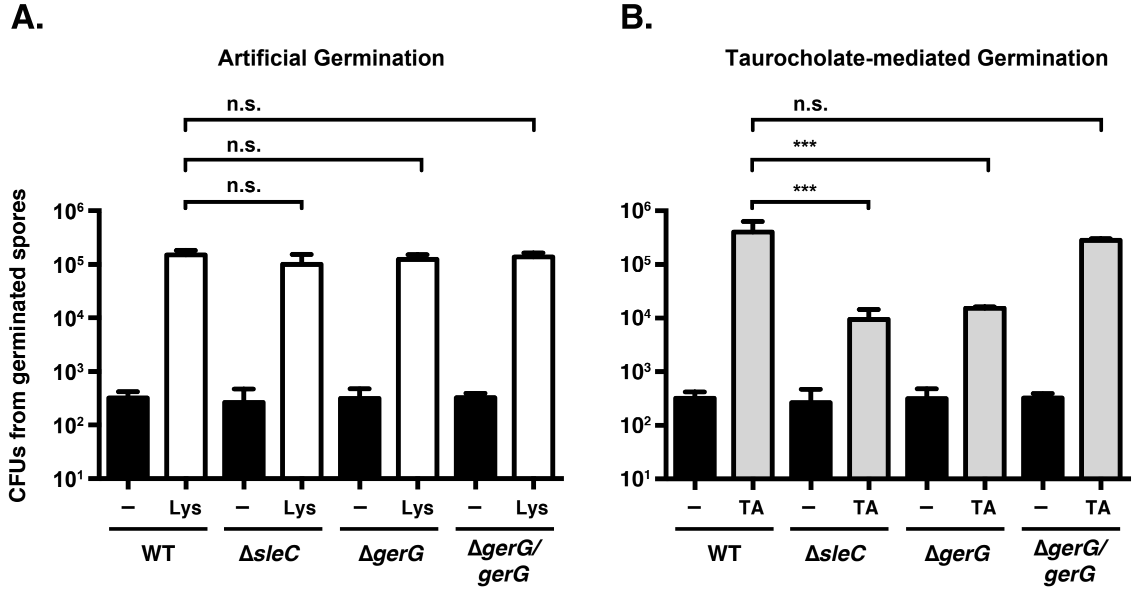

FIG S5. Artificial germination bypasses the need for GerG. (A) Wildtype (630Δ*erm*-p), Δ*sleC*, Δ*gerG*, and Δ*gerG*/*gerG* spores were either mock-treated (–) or incubated with thioglycollate and lysozyme (Lys), which can rescue the germination defect of mutants defective in cortex hydrolysis (15), and plated on BHIS (artificial germination). (B) Untreated spores used in (A) were also plated on either BHIS (–) or BHIS containing 0.5% taurocholate (TA). Data represents the average of three biological replicates. No statistically significant difference was observed between Δ*sleC* and Δ*gerG* strains subjected to artificial germination relative to wild type, in contrast with taurocholate-mediated germination. Statistical significance was determined using ANOVA and Tukey's test (*** $p < 0.001$).
