# Supplementary material for: A Clostridium difficile-Specific, Gel-Forming Protein Required for Optimal Spore Germination
Source: mBio. 2017 Jan 17;8(1):e02085-16. doi: 10.1128/mBio.02085-16 (PMC5241399; doi:10.1128/mBio.02085-16)
Supplement: FIG S6 [file mbo002173148sf6.pdf]

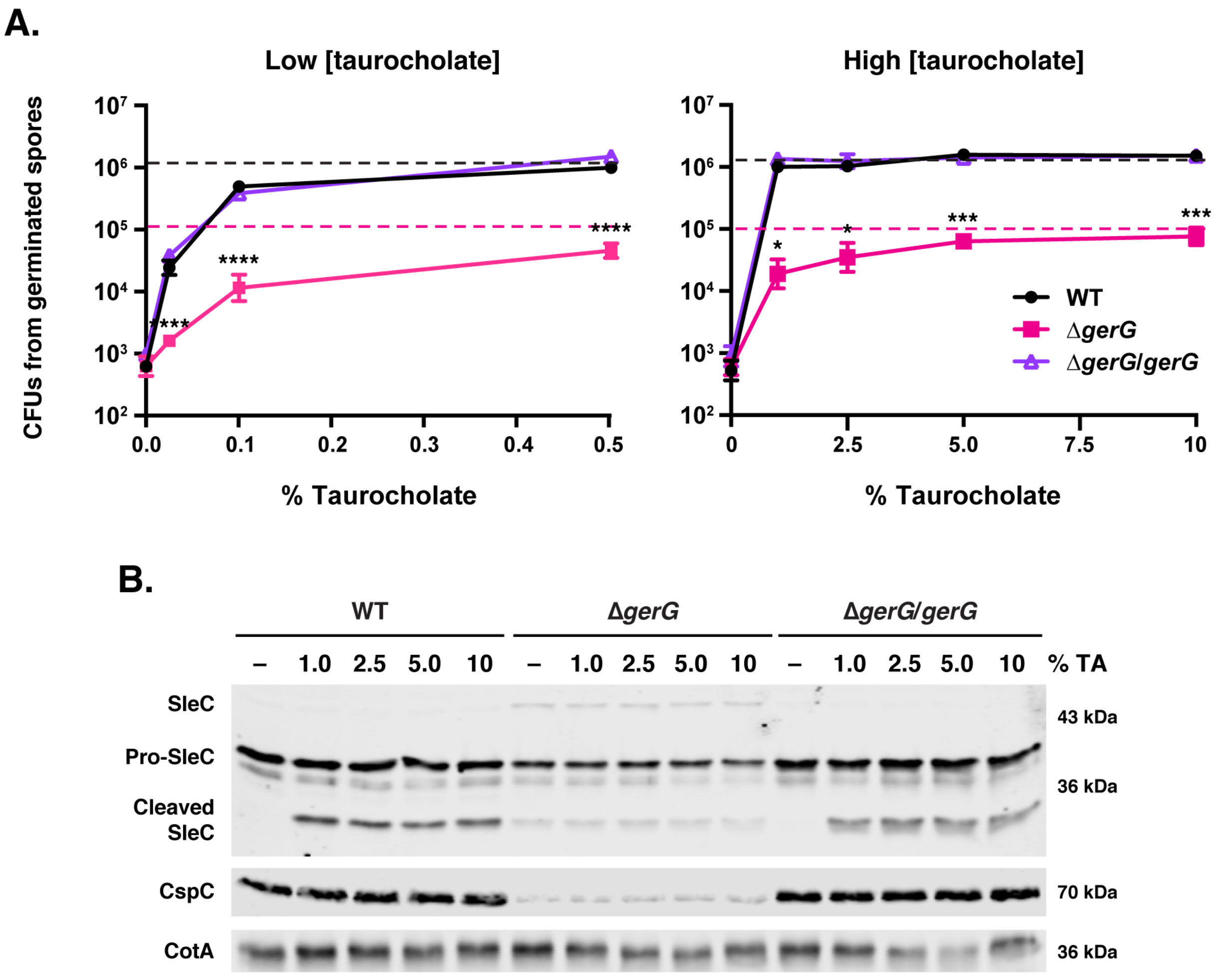

FIG S6. Responsiveness of *gerG* mutant spores to germinant. (A) Colony forming units (CFUs) that arose from wildtype (630Δ*erm*-p), Δ*gerG*, and Δ*gerG*/*gerG* spores exposed to either water (–) or increasing concentrations of taurocholate (TA) germinant for 20 min at 37˚C and plated on BHIS. The "low" TA concentrations used were 0.025% (0.5 mM), 0.1% (1.9 mM), and 0.5% (9 mM). The "high" TA concentrations used were 1% (19 mM), 2.5% (47 mM), 5% (93 mM), and 10% (186 mM). The dashed line represents the average number of colonies obtained when untreated wildtype and Δ*gerG* spores were plated on BHIS plates containing 0.1% TA. This line represents the "maximal" amount of germination that can be detected for spores of a given strain, since they are continuously exposed to germinant during the germination assay. The

“maximal” number of CFUs obtained from Δ*gerG*/*gerG* spores is not shown, but it is unchanged relative to wild type. Statistical significance relative to wild type was determined using one-way ANOVA and Tukey’s test. **** $p < 0.0001$, *** $p < 0.0005$, * $p <0.05$. The difference in colonies observed when wildtype and Δ*gerG* spores were plated on BHIS plates containing TA was statistically significant for both the low and high TA germinant titrations (i.e. comparison of pink to black dashed lines, $p < 0.0001$). (B) Western blot analyses of spores exposed to high concentrations of taurocholate (TA). SleC is detected in three major forms: full-length (SleC), zymogen (pro-SleC), and proteolytically activated (cleaved SleC, (9)). No change in SleC processing was observed for Δ*gerG* spores in response to high TA exposure, in contrast with wildtype and Δ*gerG*/*gerG* spores. CotA was used as a loading control.
