# Supplementary material for: A Clostridium difficile-Specific, Gel-Forming Protein Required for Optimal Spore Germination
Source: mBio. 2017 Jan 17;8(1):e02085-16. doi: 10.1128/mBio.02085-16 (PMC5241399; doi:10.1128/mBio.02085-16)
Supplement: FIG S7 [file mbo002173148sf7.pdf]

```
CD206          1 MSNKKKKDLDTSYMPYLDPANYADYITNEEIPQDELRNDPQISNGYTKIPNNQNINNLYPNNVNHYPKSSNSPNNLPNTN  80
CD196          1 MSNKKKKDLDTSYMPYLDPANYADYITNEEIPQDELRNDPQISNGYTKIPNNQNINNLYPNNVNHYPKSSNSPNNLPNTN  80
CD181          1 MSNKKKKDLDTSYMPYLDPANYADYITNEEIPQDELRNDPQISNGYTKIPNNQNINNLYPNNVNHYPKSSNSPNNLPNTN  80
CD169          1 MSNKKKKDLDTSYMPYLDPANYADYITNEEIPQDELRNDPQISNGYTKIPNNQNINNLYPNNVNHYPKSSNSPNNLPNTN  80
CD133          1 MSNKKKKDLDTSYMPYLDPANYADYITNEEIPQDELRNDPQISNGYTKIPNNQNINNLYPNNVNHYPKSSNSPNNLPNTN  80
CD47           1 MSNKKKKDLDTSYMPYLDPANYADYITNEEIPQDELRNDPQISNGYTKIPNNQNINNLYPNNVNHYPKSSNSPNNLPNTN  80
CD46           1 MSNKKKKDLDTSYMPYLDPANYADYITNEEIPQDELRNDPQISNGYTKIPNNQNINNLYPNNVNHYPKSSNSPNNLPNTN  80
CD43           1 MSNNKKKDLDTSYMPYLDPANYADYITNEEIPQDELRNDPQISNGYTKIPNNQNINNLYPNNVNHYPKSSNSPNNLPNTN  80
CD42           1 MSNKKKKDLDTSYMPYLDPANYADYITNEEIPQDELRNDPQISNGYTKIPNNQNINNLYPNNVNHYPKSSNSPNNLPNTN  80
CD41           1 MSNKKKKDLDTSYMPYLDPANYADYITNEEIPQDELRNDPQISNGYTKIPNNQNINNLYPNNVNHYPKSSNSPNNLPNTN  80
CD34           1 MSNKKKKDLDTSYMPYLDPANYADYITNEEIPQDELRNDPQISNGYTKIPNNQNINNLYPNNVNHYPKSSNSPNNLPNTN  80
CD18           1 MSNKKKKDLDTSYMPYLDPANYADYITNEEIPQDELRNDPQISNGYTKIPNNQNINNLYPNNVNHYPKSSNSPNNLPNTN  80
CD13           1 MSNKKKKDLDTSYMPYLDPANYADYITNEEIPQDELRNDPQISNGYTKIPNNQNINNLYPNNVNHYPKSSNSPNNLPNTN  80
CD9            1 MSNKKKKDLDTSYMPYLDPANYADYITNEEIPQDELRNDPQISNGYTKIPNNQNINNLYPNNVNHYPKSSNSPNNLPNTN  80
CD8            1 MSNKKKKDLDTSYMPYLDPANYADYITNEEIPQDELRNDPQISNGYTKIPNNQNINNLYPNNVNHYPKSSNSPNNLPNTN  80
CD_Y270        1 MSNKKKKDLDTSYMPYLDPANYADYITNEEIPQDELRNDPQISNGYTKIPNNQNINNLYPNNVNHYPKSSNSPNNLPNTN  80
CD_T61         1 MSNNKKKDLDTSYMPYLDPANYADYITNEEIPQDELRNDPQISNGYTKIPNNQNINNLYPNNVNHYPKSSNSPNNLPNTN  80
CD_QCD76w55    1 MSNKKKKDLDTSYMPYLDPANYADYITNEEIPQDELRNDPQISNGYTKIPNNQNINNLYPNNVNHYPKSSNSPNNLPNTN  80
CD_P53         1 MSNKKKKDLDTSYMPYLDPANYADYITNEEIPQDELRNDPQISNGYTKIPNNQNINNLYPNNVNHYPKSSNSPNNLPNTN  80
CD_P49         1 MSNKKKKDLDTSYMPYLDPANYADYITNEEIPQDELRNDPQISNGYTKIPNNQNINNLYPNNVNHYPKSSNSPNNLPNTN  80
CD_P46         1 MSNKKKKDLDTSYMPYLDPANYADYITNEEIPQDELRNDPQISNGYTKIPNNQNINNLYPNNVNHYPKSSNSPNNLPNTN  80
CD_P42         1 MSNKKKKDLDTSYMPYLDPANYADYITNEEIPQDELRNDPQISNGYTKIPNNQNINNLYPNNVNHYPKSSNSPNNLPNTN  80
CD_P30         1 MSNKKKKDLDTSYMPYLDPANYADYITNEEIPQDELRNDPQISNGYTKIPNNQNINNLYPNNVNHYPKSSNSPNNLPNTN  80
CD_P13         1 MSNKKKKDLDTSYMPYLDPANYADYITNEEIPQDELRNDPQISNGYTKIPNNQNINNLYPNNVNHYPKSSNSPNNLPNTN  80
CD_NAP08       1 MSNKKKKDLDTSYMPYLDPANYADYITNEEIPQGELRNDPQISNGYTKVPNNQNINNLYPNNINNYPKSSNSPNNLPNTN  80
CD_M120        1 MSNKKKKDLDTSYMPYLDPANYADYITNEEIPQGELRNDPQISNGYTKVPNNQNINNLYPNNINNYPKSSNSPNNLPNTN  80
CD_M68         1 MSNKKKKDLDTSYMPYLDPANYADYITNEEIPQDELRNDPQISNGYTKIPNNQNINNLYPNNVNHYPKSSNSPNNLPNTN  80
CD_LIBA5734    1 MSNKKKKDLDTSYMPYLDPANYADYITNEEIPQDELRNDPQISNGYTKIPNNQNINNLYPNNVNHYPKSSNSPNNLPNTN  80
CD_LIBA5701    1 MSNKKKKDLDTSYMPYLDPANYADYITNEEIPQDELRNDPQISNGYTKIPNNQNINNLYPNNVNHYPKSSNSPNNLPNTN  80
CD_F601        1 MSNKKKKDLDTSYMPYLDPANYADYITNEEIPQDELRNDPQISNGYTKIPNNQNINNLYPNNVNHYPKSSNSPNNLPNTN  80
CD_F548        1 MSNKKKKDLDTSYMPYLDPANYADYITNEEIPQDELRNDPQISNGYTKIPNNQNINNLYPNNVNHYPKSSNSPNNLPNTN  80
CD_F314        1 MSNKKKKDLDTSYMPYLDPANYADYITNEEIPQDELRNDPQISNGYTKIPNNQNINNLYPNNVNHYPKSSNSPNNLPNTN  80
CD_F152        1 MSNNKKKDLDTSYMPYLDPANYADYITNEEIPQDELRNDPQISNGYTKIPNNQNINNLYPNNVNHYPKSSNSPNNLPNTN  80
CD_E16         1 MSNNKKKDLDTSYMPYLDPANYADYITNEEIPQDELRNDPQISNGYTKIPNNQNINNLYPNNVNHYPKSSNSPNNLPNTN  80
CD_DA00261     1 MSNKKKKDLDTSYMPYLDPANYADYITNEEIPQDELRNDPQISNGYTKIPNNQNINNLYPNNVNHYPKSSNSPNNLPNTN  80
CD_DA00246     1 MSNKKKKDLDTSYMPYLDPANYADYITNEEIPQDELRNDPQISNGYTKIPNNQNINNLYPNNVNHYPKSSNSPNNLPNTN  80
CD_DA00211     1 MSNKKKKDLDTSYMPYLDPANYADYITNEEIPQDELRNDPQISNGYTKIPNNQNINNLYPNNVNHYPKSSNSPNNLPNTN  80
CD_DA00196     1 MSNKKKKDLDTSYMPYLDPANYADYITNEEIPQDELRNDPQISNGYTKIPNNQNINNLYPNNVNHYPKSSNSPNNLPNTN  80
CD_DA00126     1 MSNKKKKDLDTSYMPYLDPANYADYITNEEIPQDELRNDPQISNGYTKIPNNQNINNLYPNNVNHYPKSSNSPNNLPNTN  80
CD_CF5         1 MSNKKKKDLDTSYMPYLDPANYADYITNEEIPQDELRNDPQISNGYTKIPNNQNINNLYPNNVNHYPKSSNSPNNLPNTN  80
CD_BI9         1 MSNKKKKDLDTSYMPYLDPANYADYITNEEIPQDELRNDPQISNGYTKIPNNQNINNLYPNNVNHYPKSSNSPNNLPNTN  80
CD_BI1         1 MSNKKKKDLDTSYMPYLDPANYADYITNEEIPQDELRNDPQISNGYTKIPNNQNINNLYPNNVNHYPKSSNSPNNLPNTN  80
CD_824         1 MSNKKKKDLDTSYMPYLDPANYADYITNEEIPQDELRNDPQISNGYTKIPNNQNINNLYPNNVNHYPKSSNSPNNLPNTN  80
CD_630         1 MSNKKKKDLDTSYMPYLDPANYADYITNEEIPQDELRNDPQISNGYTKIPNNQNINNLYPNNVNHYPKSSNSPNNLPNTN  80
                 *** ***************************** **************.*************.*.***************

CD206         81 NTSGNMNYNTSATNNMPNNMNNNTGMPNNMSNNMNSNMGMPNN------MPNNMNSNMGMPNNMPNNMNSNMGMPNNVPN 154
CD196         81 NTSGNMNYNTSATNNMPNNMNNNTGMPNNMSNNMNSNMGMPNN------MPNNMNSNMGMPNNMSNNMNSNMGMPNNMPN 154
CD181         81 NTSGNMNYNTSATNNMPNNMNNNTGMPNNMSNNMNSNMGMPNN------MPNNMNSNMGMPNNMPNNMNSNMGMPN---- 150
CD169         81 NTSGNMNYNTSATNNMPNNMNNNTGMPNNMSNNMNSNMGMPNN------MSNNMNSNMGMPNNMPNNMNSNMGMPNNVPN 154
CD133         81 NTSGNMNYNTSATNNMPNNMNNNTGMPNNMSNNMNSNMGMPNN------MPNNMNSNMGMPNNMPNNMNSNMGIPNNVPN 154
CD47          81 NTSGNMNYNTSATNNMPNNMNNNTGMPNNMSNNMNSNMGMPNN------MPNNMNSNMGMPNNMPNNMNSNMGMPN---- 150
CD46          81 NTSGNMNYNTSATNNMPNNMNNNTGMPNNMSNNMNSNMGMPNN------MPNNMNSNMGMPNNMPNNMNSNMGMPNNVPN 154
CD43          81 NTSGNMNYNTSATNNMPNNMNNNTGMPNNMPNNMNSNMGMPNN--------------------MPNNMNSNMGMPNNVPN 140
CD42          81 NTSGNMNYNTSATNNMPNNMNNNTGMPNNMSNNMNSNMGMPNN------MSNNMNSNMGMPNNMPNNMNSNMGMPNNVPN 154
CD41          81 NTSGNMNYNTSATNNMPNNMNNNTGMPNNMSNNMNSNMGMPNN----------------------------------VPN 126
CD34          81 NTSGNMNYNTSATNNMPNNMNNNTGMPNNMSNNMNSNMGMPNN------MSNNMNSNMGMPNNMPNNMNSNMGMPNNVPN 154
CD18          81 NTSGNMNYNTSATNNMPNNMNNNTGMPNNMSNNMNSNMGMPNN------MSNNINSNMGMPNNMPNNMNSNMGMPNNVPN 154
CD13          81 NTSGNMNYNTSATNNMPNNMNNNTGMPNNMSNNMNSNMGMPNN------MSNNMNSNMGMPNNMPNNMNSNMGMPNNVPN 154
CD9           81 NTSGNMNYNTSATNNMPNNMNNNTGMPNNMSNNMNSNMGMPNN------MSNNINSNMGMPNNMPNNMNSNMGMPNNVPN 154
CD8           81 NTSGNMNYNTSATNNMPNNMNNNTGMPNNMSNNMNSNMGMPNN------MSNNINSNMGMPNNMPNNMNSNMGMPNNVPN 154
CD_Y270       81 NTSGNMNYNTSATNNMPNNMNNNTGMPNNMSNNMNSNMGMPNN------MPNNMNSNMGMPNNMPNNMNSNMGMPNNVPN 154
CD_T61        81 NTSGNMNYNTSATNNMPNNMNNNTGMPNNMPNNMNSNMGMPNN--------------------MPNNMNSNMGMPNNVPN 140
CD_QCD76w55   81 NTSGNMNYNTSATNNMPNNMNNNTGMPNNMSNNMNSNMGMPNN------MPNNMNSNMGMPNNMSNNMNSNMGMPNNMPN 154
CD_P53        81 NTSGNMNYNTSATNNMPNNMNNNTGMPNNMSNNMNSNMGMPNN------MPNNMNSNMGMPNNMPNNMNSNMGMPNNVPN 154
CD_P49        81 NTSGNMNYNTSATNNMPNNMNNNTGMPNNMSNNMNSNMGMPNN------MPNNMNSNMGMPNNMPNNMNSNMGMPNNVPN 154
CD_P46        81 NTSGNMNYNTSATNNMPNNMNNNTGMPNNMSNNMNSNMGMPNN------MSNNINSNMGMPNNMPNNMNSNMGMPNNVPN 154
CD_P42        81 NTSGNMNYNTSATNNMPNNMNNNTGMPNNMSNNMNSNMGMPNN------MPNNMNSNMGMPNNMPNNMNSNMGMPNNVPN 154
CD_P30        81 NTSGNMNYNTSATNNMPNNMNNNTGMPNNMSNNMNSNMGMPNN------MPNNMNSNMGMPNNMPNNMNSNMGMPNNVPN 154
CD_P13        81 NTSGNMNYNTSATNNMPNNMNNNTGMPNNMSNNMNSNMGMPNN------MSNNINSNMGMPNNMPNNMNSNMGMPNNVPN 154
CD_NAP08      81 NTSGNMNYNTSATNNMPNNMNSNMGTPNNMSNNMNSNMGIPNN------MPNNMNSNMGIPNNMPNNMNSNMGIPNNMPN 154
CD_M120       81 NTSGNMNYNTSATNNMPNNMNSNMGTPNNMSNNMNSNMGIPNN------MPNNMNSNMGIPNNMPNNMNSNMGIPNNMPN 154
CD_M68        81 NTSGNMNYNTSATNNMPNNMNNNTGMPNNMSNNMNSNMGMPNNMNSNMGMPNNMNSNMGMPNNMPNNMNSNMGMPN---- 156
CD_LIBA5734   81 NTSGNMNYNTSATNNMPNNMNNNTGMPNNMSNNMNSNMGMPNN------MSNNMNSNMGMPNNMPNNMNSNMGMPNNVPN 154
CD_LIBA5701   81 NTSGNMNYNTSATNNMPNNMNNNTGMPNNMSNNMNSNMGMPNN------MSNNMNSNMGMPNNMPNNMNSNMGMPNNVPN 154
CD_F601       81 NTSGNMNYNTSATNNMPNNMNNNTGMPNNMSNNMNSNMGMPNN------MPNNMNSNMGMPNNMPNNMNSNMGMPNNVPN 154
CD_F548       81 NTSGNMNYNTSATNNMPNNMNNNTGMPNNMSNNMNSNMGMPNN------MSNNMNSNMGMPNNMPNNMNSNMGMPNNVPN 154
CD_F314       81 NTSGNMNYNTSATNNMPNNMNNNTGMPNNMSNNMNSNMGMPNN------MPNNMNSNMGMPNNMPNNMNSNMGMPNNMPN 154
CD_F152       81 NTSGNMNYNTSATNNMPNNMNNNTGMPNNMPNNMNSNMGMPNN--------------------MPNNMNSNMGMPNNVPN 140
CD_E16        81 NTSGNMNYNTSATNNMPNNMNNNTGMPNNMPNNMNSNMGMPNN--------------------MPNNMNSNMGMPNNVPN 140
CD_DA00261    81 NTSGNMNYNTSATNNMPNNMNNNTGMPNNMSNNMNSNMGMPNN------MSNNMNSNMGMPNNMPNNMNSNMGMPNNVPN 154
```

```
CD_DA00246      81 NTSGNMNYNTSATNNMPNNMNNNTGMPNNMSNNMNSNMGMPNN------MPNNMNSNMGMPNNMPNNMNSNMGIPNNVPN 154
CD_DA00211      81 NTSGNMNYNTSATNNMPNNMNNNTGMPNNMSNNMNSNMGMPNN------MPNNMNSNMGMPNNMPNNMNSNMGMPNNVPN 154
CD_DA00196      81 NTSGNMNYNTSATNNMPNNMNNNTGMPNNMSNNMNSNMGMPNN------MPNNMNSNMGMPNNMPNNMNSNMGMPNNVPN 154
CD_DA00126      81 NTSGNMNYNTSATNNMPNNMNNNTGMPNNMSNNMNSNMGMPNN------MPNNMNSNMGMPNNMPNNMNSNMGMPNNVPN 154
CD_CF5          81 NTSGNMNYNTSATNNMPNNMNNNTGMPNNMSNNMNSNMGMPNNMNSNMGMPNNMNSNMGMPNNMPNNMNSNMGMPN---- 156
CD_BI9          81 NTSGNMNYNTSATNNMPNNMNNNTGMPNNMSNNMNSNMGMPNN------MPNNMNSNMGMPNNMPNNMNSNMGMPN---- 150
CD_BI1          81 NTSGNMNYNTSATNNMPNNMNNNTGMPNNMSNNMNSNMGMPNN------MPNNMNSNMGMPNNMSNNMNSNMGMPNNMPN 154
CD_824          81 NTSGNMNYNTSATNNMPNNMNNNTGMPNNMSNNMNSNMGMPNN------MSNNMNSNMGMPNNMPNNMNSNMGMPNNVPN 154
CD_630          81 NTSGNMNYNTSATNNMPNNMNNNTGMPNNMSNNMNSNMGMPNN------MSNNMNSNMGMPNNMPNNMNSNMGMPNNVPN 154
                     ********************* * * **** ********.***

CD206           155 NMNSNMGMPNNMSNNMNSNMGMPNNMS--------------GNMNNNMYGNTGMPNNMNSNMGYNMNNTMGIPNFSCNQN 220
CD196           155 NMNSNMGMPNNMPNNMNSNMGMPNNMP--------------NNMNSNMGMPNNVPNNMNSNMGYNMNNTMGIPNFSCNQN 220
CD181           151 NMNSNMGIPNNMSNNMNSNMGMPNNMS--------------GNM-NNMYGNTSMPNNMNSNMGYNMNNTMSIPNFSCNQN 215
CD169           155 NMNSNMGMPNNMSNNMNSNMGMPNNMS--------------GNMNNNMYGNTGMPNNMNSNMGYNMNNTMGIPNFSCNQN 220
CD133           155 NMNSNMGMPNNMSNNMNSNMGMPNNMS--------------GNMNNNMYGNTGMPNNMNSNMGYNMNNTMGIPNFSCNQN 220
CD47            151 NMNSNMGIPNNMSNNMNSNMGMPNNMS--------------GNM-NNMYGNTSMPNNMNSNMGYNMNNTMSIPNFSCNQN 215
CD46            155 NMNSNMGMPNNMSNNMNSNMGMPNNMS--------------GNMNNNMYGNTGMPNNMNSNMGYNMNNTMGIPNFSCNQN 220
CD43            141 NMNSNMGMPNNMSNNMNSNMGMPNNMS--------------GNM-NNMYGDTGMPNNMNSNMGYNMNNTMGIPNFSCNQN 205
CD42            155 NMNSNMGMPNNMSNNMNSNMGMPNNMS--------------GNMNNNMYGNTGMPNNMNSNMGYNMNNTMGIPNFSCNQN 220
CD41            127 NMNSNMGMPNNMSNNMNSNMGMPNNMS--------------GNMNNNMYGNTGMPNNMNSNMGYNMNNTMGIPNFSCNQN 192
CD34            155 NMNSNMGMPNNMSNNMNSNMGMPNNMS--------------GNMNNNMYGNTGMPNNMNSNMGYNMNNTMGIPNFSCNQN 220
CD18            155 NMNSNMGMPNNISNNMNSNMGMPNNMS--------------GNMNNNMYGNTGMPNNMNSNMGYNMNNTMGIPNFSCNQN 220
CD13            155 NMNSNMGMPNNMSNNMNSNMGMPNNMS--------------GNMNNNMYGNTGMPNNMNSNMGYNMNNTMGIPNFSCNQN 220
CD9             155 NMNSNMGMPNNISNNMNSNMGMPNNMS--------------GNMNNNMYGNTGMPNNMNSNMGYNMNNTMGIPNFSCNQN 220
CD8             155 NMNSNMGMPNNISNNMNSNMGMPNNMS--------------GNMNNNMYGNTGMPNNMNSNMGYNMNNTMGIPNFSCNQN 220
CD_Y270         155 NMNSNMGMPNNISNNMNSNMGMPNNMS--------------GNMNNNMYGNTGMPNNMNSNMGYNMNNTMGIPNFSCNQN 220
CD_T61          141 NMNSNMGMSNNMSNNMNSNMGMPNNMS--------------GNM-NNMYGDTGMPNNMNSNMGYNMNNTMGIPNFSCNQN 205
CD_QCD76w5      155 NMNSNMGMPNNMPNNMNSNMGMPNNMP--------------NNMNSNMGMPNNVPNNMNSNMGYNMNNTMGIPNFSCNQN 220
CD_P53          155 NMNSNMGMPNNMSNNMNSNMGMPNNMS--------------GNMNNNMYGNTGMPNNMNSNMGYNMNNTMGIPNFSCNQN 220
CD_P49          155 NMNSNMGMPNNMSNNMNSNMGMPNNMS--------------GNMNNNMYGNTGMPNNMNSNMGYNMNNTMGIPNFSCNQN 220
CD_P46          155 NMNSNMGMPNNISNNMNSNMGMPNNMS--------------GNMNNNMYGNTGMPNNMNSNMGYNMNNTMGIPNFSCNQN 220
CD_P42          155 NMNSNMGMPNNMSNNMNSNMGMPNNMS--------------GNMNNNMYGNTGMPNNMNSNMGYNMNNTMGIPNFSCNQN 220
CD_P30          155 NMNSNMGMPNNMSNNMNSNMGMPNNMS--------------GNMNNNMYGNTGMPNNMNSNMGYNMNNTMGIPNFSCNQN 220
CD_P13          155 NMNSNMGMPNNISNNMNSNMGMPNNMS--------------GNMNNNMYGNTGMPNNMNSNMGYNMNNTMGIPNFSCNQN 220
CD_NAP08        155 NMNSNMGIPNNMPNNMNSNMGIPNNMY--------------GNMNNNMYGNTGMPNNMNSNMGYNMNNTMGIPNFSCNQN 220
CD_M120         155 NMNSNMGIPNNMPNNMNSNMGIPNNMPNNMNSNMGIPNNMYGNMNNNMYGNTGMPNNMNSNMGYNMNNTMGIPNFSCNQN 234
CD_M68          157 NMNSNMGIPNNMSNNMNSNMGMPNNMS--------------GNM-NNMYGNTSMPNNMNSNMGYNMNNTMGIPNFSCNQN 221
CD_LIBA5734     155 NMNSNMGMPNNMSNNMNSNMGMPNNMS--------------GNMNNNMYGNTGMPNNMNSNMGYNMNNTMGIPNFSCNQN 220
CD_LIBA5701     155 NMNSNMGMPNNMSNNMNSNMGMPNNMS--------------GNMNNNMYGNTGMPNNMNSNMGYNMNNTMGIPNFSCNQN 220
CD_F601         155 NMNSNMGMPNNMSNNMNSNMGMPNNMS--------------GNMNNNMYGNTGMPNNMNSNMGYNMNNTMGIPNFSCNQN 220
CD_F548         155 NMNSNMGMPNNMSNNMNSNMGMPNNMS--------------GNMNNNMYGNTGMPNNMNSNMGYNMNNTMGIPNFSCNQN 220
CD_F314         155 NMNSNMGMPNN------------------------------------------VPNNMNSNMGYNMNNTMGIPNFSCNQN 192
CD_F152         141 NMNSNMGMPNNMSNNMNSNMGMPNNMS--------------GNM-NNMYGDTGMPNNMNSNMGYNMNNTMGIPNFSCNQN 205
CD_E16          141 NMNSNMGMPNNMSNNMNSNMGMPNNMS--------------GNM-NNMYGDTGMPNNMNSNMGYNMNNTMGIPNFSCNQN 205
CD_DA00261      155 NMNSNMGMPNNMSNNMNSNMGMPNNMS--------------GNMNNNMYGNTGMPNNMNSNMGYNMNNTMGIPNFSCNQN 220
CD_DA00246      155 NMNSNMGMPNNMSNNMNSNMGMPNNMS--------------GNMNNNMYGNTGMPNNMNSNMGYNMNNTMGIPNFSCNQN 220
CD_DA00211      155 NMNSNMGMPNNMSNNMNSNMGMPNNMS--------------GNM-NNMYGNTGMPNNMNSNMGYNMNNTMGIPNFSCNQN 219
CD_DA00196      155 NMNSNMGMPNNISNNMNSNMGMPNNMS--------------GNMNNNMYGNTGMPNNMNSNMGYNMNNTMGIPNFSCNQN 220
CD_DA00126      155 NMNSNMGMPNNMSNNMNSNMGMPNNMS--------------GNMNNNMYGNTGMPNNMNSNMGYNMNNTMGIPNFSCNQN 220
CD_CF5          157 NMNSNMGIPNNMSNNMNSNMGMPNNMS--------------GNM-NNMYGNTSMPNNMNSNMGYNMNNTMGIPNFSCNQN 221
CD_BI9          151 NMNSNMGIPNNMSNNMNSNMGMPNNMS--------------GNM-NNMYGNTSMPNNMNSNMGYNMNNTMSIPNFSCNQN 215
CD_BI1          155 NMNSNMGMPNNMPNNMNSNMGMPNNMP--------------NNMNSNMGMPNNVPNNMNSNMGYNMNNTMGIPNFSCNQN 220
CD_824          155 NMNSNMGMPNNMSNNMNSNMGMPNNMS--------------GNMNNNMYGNTGMPNNMNSNMGYNMNNTMGIPNFSCNQN 220
CD_630          155 NMNSNMGMPNNMSNNMNSNMGMPNNMS--------------GNMNNNMYGNTGMPNNMNSNMGYNMNNTMGIPNFSCNQN 220
                     *******. **                                          .**************** *********

CD206           221 MPPNVLMMIPGVICHNTMQGMPVVMPSTMPPNIYPTPYGSSNMSVQGIPQATNIEEFDEEEM 282
CD196           221 MPPNVLMMIPGVICHNTMQGMPVVMPSTMPPNIYPTPYGSSNMSIQGIPQATNIEEFDEEEM 282
CD181           216 MPPNVLMMIPGVICHNTMQGMPVVMPSTMPPNIYPTPYGSSNMSIQGIPQTTNIEEFDEEEM 277
CD169           221 MPPNVLMMIPGVICHNTMQGMPVVMPSTMPPNIYPTPYGSSNMSIQGIPQATNIEEFDEEEM 282
CD133           221 MPPNVLMMIPGVICHNTMQGMPVVMPSTMPPNIYPTPYGSSNMSIQGIPQATNIEEFDEEEM 282
CD47            216 MPPNVLMMIPGVICHNTMQGMPVVMPSTMPPNIYPTPYGSSNMSIQGIPQTTNIEEFDEEEM 277
CD46            221 MPPNVLMMIPGVICHNTMQGMPVVMPSTMPPNIYPTPYGSSNMSVQGIPQATNIEEFDEEEM 282
CD43            206 MPPNVLMMIPGVICHNTMQGMPVVMPSTMPPNIYTTPYGSSNMSTQGIPQTTNIEEFDEEEM 267
CD42            221 MPPNVLMMIPGVICHNTMQGMPVVMPSTMPPNIYPTPYGSSNMSIQGIPQATNIEEFDEEEM 282
CD41            193 MPPNVLMMIPGVICHNTMQGMPVVMPSTMPPNIYPTPYGSSNMSVQGIPQATNIEEFDEEEM 254
CD34            221 MPPNVLMMIPGVICHNTMQGMPVVMPSTMPPNIYPTPYGSSNMSVQGIPQATNIEEFDEEEM 282
CD18            221 MPPNVLMMIPGVICHNTMQGMPVVMPSTMPPNIYPTPYGSSNMSIQGIPQATNIEEFDEEEM 282
CD13            221 MPPNVLMMIPGVICHNTMQGMPVVMPSTMPPNIYPTPYGSSNMSIQGIPQATNIEEFDEEEM 282
CD9             221 MPPNVLMMIPGVICHNTMQGMPVVMPSTMPPNIYPTPYGSSNMSIQGIPQATNIEEFDEEEM 282
CD8             221 MPPNVLMMIPGVICHNTMQGMPVVMPSTMPPNIYPTPYGSSNMSIQGIPQATNIEEFDEEEM 282
CD_Y270         221 MPPNVLMMIPGVICHNTMQGMPVVMPSTMPPNIYPTPYGSSNMSVQGIPQATNIEEFDEEEM 282
CD_T61          206 MPPNVLMMIPGVICHNTMQGMPVVMPSTMPPNIYPTPYGSSNMSVQGIPQATNIEEFDEEEM 267
CD_QCD76w55     221 MPPNVLMMIPGVICHNTMQGMPVVMPSTMPPNIYPTPYGSSNMSIQGIPQATNIEEFDEEEM 282
CD_P53          221 MPPNVLMMIPGVICHNTMQGMPVVMPSTMPPNIYPTPYGSSNMSIQGIPQATNIEEFDEEEM 282
CD_P49          221 MPPNVLMMIPGVICHNTMQGMPVVMPSTMPPNIYPTPYGSSNMSIQGIPQATNIEEFDEEEM 282
CD_P46          221 MPPNVLMMIPGVICHNTMQGMPVVMPSTMPPNIYPTPYGSSNMSIQGIPQATNIEEFDEEEM 282
CD_P42          221 MPPNVLMMIPGVICHNTMQGMPVVMPSTMPPNIYPTPYGSSNMSIQGIPQATNIEEFDEEEM 282
CD_P30          221 MPPNVLMMIPGVICHNTMQGMPVVMPSTMPPNIYPTPYGSSNMSIQGIPQATNIEEFDEEEM 282
CD_P13          221 MPPNVLMMIPGVICHNTMQGMPVVMPSTMPPNIYPTPYGSSNMSIQGIPQATNIEEFDEEEM 282
```

```
CD_NAP08       221 MPPNVLMMIPGVICHNTMQGMPVVMPSTMPPNIYPTPYGSSNMSMQGIPQATNIEEFDEEEM 282
CD_M120        235 MPPNVLMMIPGVICHNTMQGMPVVMPSTMPPNIYPTPYGSSNMSMQGIPQATNIEEFDEEEM 296
CD_M68         222 MPPNVLMMIPGVICHNTMQGMPVVMPSTMPPNIYPTPYGSSNMSIQGIPQTTNIEEFDEEEM 283
CD_LIBA5734    221 MPPNVLMMIPGVICHNTMQGMPVVMPSTMPPNIYPTPYGSSNMSIQGIPQATNIEEFDEEEM 282
CD_LIBA5701    221 MPPNVLMMIPGVICHNTMQGMPVVMPSTMPPNIYPTPYGSSNMSIQGIPQATNIEEFDEEEM 282
CD_F601        221 MPPNVLMMIPGVICHNTMQGMPVVMPSTMPPNIYPTPYGSSNMSIQGIPQATNIEEFDEEEM 282
CD_F548        221 MPPNVLMMIPGVICHNTMQGMPVVMPSTMPPNIYPTPYGSSNMSVQGIPQATNIEEFDEEEM 282
CD_F314        193 MPPNVLMMIPGVICHNTMQGMPVVMPSTMPPNIYPTPYGSSNMSIQGIPQATNIEEFDEEEM 254
CD_F152        206 MPPNVLMMIPGVICHNTMQGMPVVMPSTMPPNIYTTPYGSSNMSTQGIPQTTNIEEFDEEEM 267
CD_E16         206 MPPNVLMMIPGVICHNTMQGMPVVMPSTMPPNIYPTPYGSSNMSVQGIPQATNIEEFDEEEM 267
CD_DA00261     221 MPPNVLMMIPGVICHNTMQGMPVVMPSTMPPNIYPTPYGSSNMSIQGIPQATNIEEFDEEEM 282
CD_DA00246     221 MPPNVLMMIPGVICHNTMQGMPVVMPSTMPPNIYPTPYGSSNMSIQGIPQATNIEEFDEEEM 282
CD_DA00211     220 MPPNVLMMIPGVICHNTMQGMPVVMPSTMPPNIYPTPYVSSNMSVQGIPQATNIEEFDEEEM 281
CD_DA00196     221 MPPNVLMMIPGVICHNTMQGMPVVMPSTMPPNIYPTPYGSSNMSVQGIPQATNIEEFDEEEM 282
CD_DA00126     221 MPPNVLMMIPGVICHNTMQGMPVVMPSTMPPNIYPTPYGSSNMSIQGIPQATNIEEFDEEEM 282
CD_CF5         222 MPPNVLMMIPGVICHNTMQGMPVVMPSTMPPNIYPTPYGSSNMSIQGIPQTTNIEEFDEEEM 283
CD_BI9         216 MPPNVLMMIPGVICHNTMQGMPVVMPSTMPPNIYPTPYGSSNMSIQGIPQTTNIEEFDEEEM 277
CD_BI1         221 MPPNVLMMIPGVICHNTMQGMPVVMPSTMPPNIYPTPYGSSNMSIQGIPQATNIEEFDEEEM 282
CD_824         221 MPPNVLMMIPGVICHNTMQGMPVVMPSTMPPNIYPTPYGSSNMSVQGIPQATNIEEFDEEEM 282
CD_630         221 MPPNVLMMIPGVICHNTMQGMPVVMPSTMPPNIYPTPYGSSNMSIQGIPQATNIEEFDEEEM 282
                   ********************************** *** ***** *****.***********
```

Fig. S7. ClustalW alignment of expanded GerG homologs. Identical repeat sequences are colored as either red, green, blue or purple. Maroon-colored repeat sequences are identical to the repeat sequences shown in red. Dark green-colored repeat sequences are identical to the repeat sequences shown in green.
