# Supplementary material for: A Clostridium difficile-Specific, Gel-Forming Protein Required for Optimal Spore Germination
Source: mBio. 2017 Jan 17;8(1):e02085-16. doi: 10.1128/mBio.02085-16 (PMC5241399; doi:10.1128/mBio.02085-16)
Supplement: FIG S9 [file mbo002173148sf9.pdf]

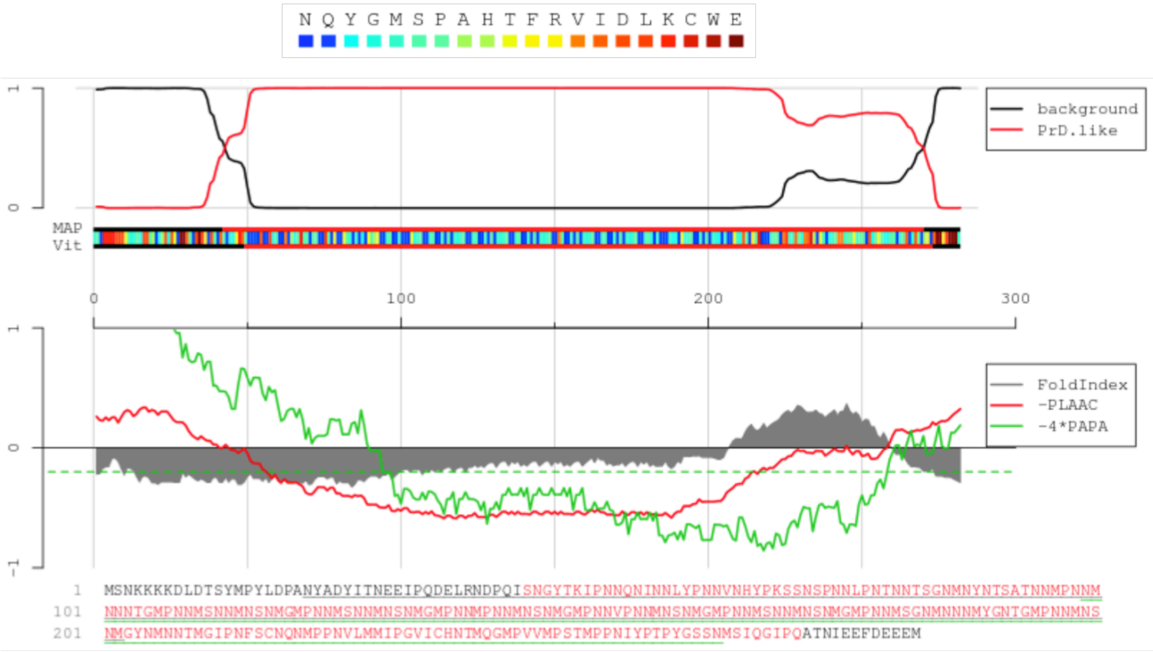

FIG S9. PLAAC analysis of GerG. Predicted prion-like domains (PrDs) in GerG are highlighted in red. Each amino acid is color coded by its enrichment log-likelihood ratio in PrDs. 4*PAPA represents PrD prediction using the PAPA algorithm, while FoldIndex predicts intrinsically unfolded protein regions (16).
