# Supplementary material for: A Clostridium difficile-Specific, Gel-Forming Protein Required for Optimal Spore Germination
Source: mBio. 2017 Jan 17;8(1):e02085-16. doi: 10.1128/mBio.02085-16 (PMC5241399; doi:10.1128/mBio.02085-16)
Supplement: TEXT S1 [file mbo002173148s1.pdf]

Supplemental Materials

Supplemental Materials and Methods
Strain Construction, Artificial Germination, Antibody Production, and Western blot analyses

Supplemental References

Supplemental Tables
Tables S1-S6 are available at the following link:
https://drive.google.com/file/d/0B0M1PLMSo_vDb24yTXNKVFZQaTA/view?usp=sharing

Table S1. Spore purification yields for Δ*gerG* strains relative to wild type.

Table S2. Germination of *sleC* mutants in 630Δ*erm* and JIR8094 over time on rich media containing taurocholate.

Table S3. *sleC* mutant germination varies between spore preparations.

Table S4. Single spore germination analyses of Δ*gerG* in the presence of increasing concentrations of taurocholate at 37˚C.

Table S5. Concentration-dependent gel formation by recombinant GerG variants over time.

Table S6. Strains and plasmids used in this study.

Table S7. Primers used in this study.
https://drive.google.com/file/d/0B0M1PLMSo_vDVzk4Y3RZdVFSbW8/view?usp=sharing

Supplemental References

1. **Francis MB, Allen CA, Shrestha R, Sorg JA.** 2013. Bile acid recognition by the *Clostridium difficile* germinant receptor, CspC, is important for establishing infection. PLoS Pathog **9:**e1003356.
2. **Horton R, Hunt H, Ho S, Pullen J, Pease L.** 1989. Engineering hybrid genes without the use of restriction enzymes: gene splicing by overlap extension. Gene **77:**61-68.
3. **Gibson DG, Young L, Chuang RY, Venter JC, Hutchison CA, 3rd, Smith HO.** 2009. Enzymatic assembly of DNA molecules up to several hundred kilobases. Nat Methods **6:**343-345.
4. **Fimlaid KA, Bond JP, Schutz KC, Putnam EE, Leung JM, Lawley TD, Shen A.** 2013. Global Analysis of the Sporulation Pathway of *Clostridium difficile*. PLoS Genet **9:**e1003660.
5. **Heap JT, Pennington OJ, Cartman ST, Carter GP, Minton NP.** 2007. The ClosTron: a universal gene knock-out system for the genus *Clostridium*. J Microbiol Methods **70:**452-464.
6. **Ng YK, Ehsaan M, Philip S, Collery MM, Janoir C, Collignon A, Cartman ST, Minton NP.** 2013. Expanding the repertoire of gene tools for precise manipulation of the *Clostridium difficile* genome: allelic exchange using *pyrE* alleles. PLoS One **8:**e56051.
7. **Fimlaid KA, Jensen O, Donnelly ML, Francis MB, Sorg JA, Shen A.** 2015. Identification of a Novel Lipoprotein Regulator of *Clostridium difficile* Spore Germination. PLoS Pathogens doi:10.1371/journal.ppat.1005239.
8. **Putnam EE, Nock AM, Lawley TD, Shen A.** 2013. SpoIVA and SipL are *Clostridium difficile* spore morphogenetic proteins. J Bacteriol **195:**1214-1225.
9. **Adams CM, Eckenroth BE, Putnam EE, Doublie S, Shen A.** 2013. Structural and functional analysis of the CspB protease required for *Clostridium* spore germination. PLoS Pathog **9:**e1003165.
10. **Donnelly ML, Fimlaid KA, Shen A.** 2016. Characterization of *Clostridium difficile* Spores Lacking Either SpoVAC or Dipicolinic Acid Synthetase. J Bacteriol **198:**1694-1707.
11. **Fimlaid KA, Jensen O, Donnelly ML, Siegrist MS, Shen A.** 2015. Regulation of *Clostridium difficile* Spore Formation by the SpoIIQ and SpoIIIA Proteins. PLoS Genet **11:**e1005562.
12. **Kevorkian Y, Shirley DJ, Shen A.** 2015. Regulation of *Clostridium difficile* spore germination by the CspA pseudoprotease domain. Biochimie doi:10.1016/j.biochi.2015.07.023.
13. **Kong L, Zhang P, Wang G, Yu J, Setlow P, Li YQ.** 2011. Characterization of bacterial spore germination using phase-contrast and fluorescence microscopy, Raman spectroscopy and optical tweezers. Nat Protoc **6:**625-639.
14. **Deakin LJ, Clare S, Fagan RP, Dawson LF, Pickard DJ, West MR, Wren BW, Fairweather NF, Dougan G, Lawley TD.** 2012. The *Clostridium difficile spo0A* gene is a persistence and transmission factor. Infect Immun **80:**2704-2711.
15. **Kamiya S, Yamakawa K, Ogura H, Nakamura S.** 1989. Recovery of spores of *Clostridium difficile* altered by heat or alkali. J Med Microbiol **28:**217-221.

16. **Lancaster AK, Nutter-Upham A, Lindquist S, King OD.** 2014. PLAAC: a web and command-line application to identify proteins with prion-like amino acid composition. Bioinformatics **30:**2501-2502.
17. **Dineen SS, Villapakkam AC, Nordman JT, Sonenshein AL.** 2007. Repression of *Clostridium difficile* toxin gene expression by CodY. Mol Microbiol **66:**206-219.
18. **Heap J, Pennington O, Cartman S, Minton N.** 2009. A modular system for *Clostridium* shuttle plasmids. JMicrobiol Meth **78:**79-85.

Supplemental Materials

***E. coli* strain construction**

E. coli strains are listed in Table S5; all primers are listed in Table S6. *E.* For disruption of *CD0311* (*gerG*), a modified plasmid containing the retargeting group II intron, pCE245 (a gift from C. Ellermeier, University of Iowa), was used as the template. The following primers were used to amplify the targeting sequence from the pCE245: #1284, 1285, 1286 and 532, the EBS Universal primer (Sigma Aldrich). The resulting retargeting sequence was digested with BsrGI and HindIII and cloned into pJS107 (1), which is a derivative of pJIR750ai (Sigma Aldrich). The ligations were transformed into DH5α, and the resulting plasmids were confirmed by sequencing and then transformed into HB101/pRK24.

To construct the pMTL83151-*gerG* complementation construct, primers #1695 and 1696 were used to amplify the *gerG* gene containing 266 bp upstream of *gerG* using 630 genomic DNA as the template. To construct the pMTL83151-*sleC* complementation construct, primers #479 and 662 were used to amplify 244 bp upstream of *sleC* using 630 genomic DNA as the template. The resulting PCR fragments were digested with NotI and XhoI, ligated into pMTL83151 digested with the same enzymes, and the ligation was transformed into DH5α. The resulting plasmids were confirmed by sequencing and then transformed into HB101/pRK24.

To construct the pMTL-YN1C-*cspBAC* complementation construct, primer pair 691 and 665 was used to amplify the *cspBA-cspC* operon. The resulting PCR product was digested with NotI and XhoI and ligated to pMTL-YN1C digested with the same enzymes. To construct pMTL-YN1C complementation constructs for *gerG* and *sleC*, primer pair #2057 and 2058 and primer pair #2038 and 2039 were used to amplify the *gerG* gene with 266 bp of upstream

sequence and *sleC* with 199 bp of upstream sequence, respectively. To construct the *gerG* complementation constructs encoding deletions of repeat sequences 1-6 and 1-7, the internal SOE primers #1907 and 1908 and #1909 and 1910 were used, respectively, in PCR splicing by overlap extension (SOE) (2) reactions with the flanking primers #2057 and 2058. The resulting PCR fragments were ligated to pMTL-YN1C digested with NotI and XhoI using Gibson assembly (3). The assembly mixture was transformed into DH5α, and the resulting plasmids were confirmed by sequencing and then transformed into HB101/pRK24.

To construct the pMTL-YN3-Δ*spo0A* allelic exchange construct, primers #1861 and #1863 were used to amplify a 732 bp upstream of *spo0A*, and primers #1862 and #1864 were used to amplify a region 804 bp downstream of *spo0A* using 630 genomic DNA as the template. The resulting PCR products were used in a PCR SOE reaction with the flanking primers #1861 and #1864. The PCR SOE product fuses the first 8 codons of *spo0A* to the last 14 codons of *spo0A*; this product was digested with AscI and SbfI and ligated to pMTL-YN3 digested with the same enzymes. The ligation was transformed into DH5α, and the resulting plasmid was confirmed by sequencing and then transformed into HB101/pRK24.

To construct the pMTL-YN3 constructs encoding deletions of the *gerG*, *sleC*, and *cspBAC* operon, Gibson assembly was used. For *gerG*, the primer pair #2013 and 2012 was used to amplify 1087 bp upstream of *gerG*, and primer pair #2011 and 2014 was used to amplify a region 1001 bp downstream of *gerG*. The Δ*gerG* construct fuses the first 14 codons of *gerG* to the last 10 codons of *gerG*. For *sleC*, the primer pair #1991 and 1988 was used to amplify 955 bp upstream of *sleC*, and the primer pair #1987 and 1992 was used to amplify 936 bp downstream of *sleC*. The Δ*sleC* construct fuses the first 9 codons of *sleC* to the last 12 codons of *sleC*. For the Δ*cspBAC* construct, the primer pair #2179 and 1982 was used to amplify a 971 bp

fragment upstream of *cspBA*, and primer pair #1981 and 2180 was used to amplify a 1012 bp fragment downstream of *cspC*. The Δ*cspBAC* construct fuses the first 18 codons of *cspBA* to the last 6 codons of *cspC*.

To construct a strain for producing His_6-tagged GerG, primer pair #1256 and 1257 was used to amplify *gerG* using genomic DNA as the template. The resulting PCR products were digested with NdeI and XhoI, ligated to pET22b, and transformed into DH5α. The resulting pET22b-*gerG* plasmid encodes GerG with a C-terminal His_6-tag and was used to transform BL21(DE3) for protein production. To clone *gerG* constructs encoding C-terminally His_6-tagged GerG with internal deletions of the repeat regions, PCR SOE was used. Primers #1256 and 1257 were used as the flanking primers, while primer pairs #1796 and 1797; #1907 and 1908; and #1909 and 1910 were used to clone the Δ1-5, Δ1-6, and Δ1-7 variants, respectively. It should be noted that primer pair #1796 and 1797 was originally designed to delete only the first repeat sequence, but because of repeat sequences at the DNA level, this primer pair ended up creating a deletion of the region encoding repeats 1-5.

To construct a strain for producing His_6-tagged CotA, primer pair #881 and 882 were used to amplify *cotA* using genomic DNA as the template. The resulting PCR products were digested with NdeI and XhoI, ligated to pET21a, and transformed into DH5α. The resulting pET21a-*cotA* plasmid encodes GerG with a C-terminal His_6-tag and was used to transform BL21(DE3) for protein production.

C. difficile strain construction

<u>JIR8094 Strains</u>

C. difficile strains derived from the parent strain JIR8094 were constructed using TargeTron-based gene disruption as described previously (FIG S1, (4, 5)). Erythromycin-resistant patches were struck out for isolation onto the same media and individual colonies were screened by colony PCR for a 2 kb increase in the size of *gerG*, using primer pair #1256 and 1257, and *sleC* using primer pair #2038 ad 2039 .

630 Strains

Allele coupled exchange was used to construct clean deletions of *cspBAC*, *gerG, sleC*, and *spo0A* (6). The recipient *C. difficile* strain 630Δ*erm*Δ*pyrE* (a kind gift from Nigel Minton, c/o Marcin Dembek) was grown for 5-6 hrs in BHIS. HB101/pRK24 donor strains carrying the appropriate pMTL-YN3 allelic exchange constructs were grown in LB containing ampicillin (50 µg/mL) and chloramphenicol (20 µg/mL) at 37˚C, 225 rpm, under aerobic conditions, for 5-6 hrs. The *E. coli* strain was pelleted at 2,500 rpm for 5 min and transferred into the chamber. One milliliter of the *C. difficile* culture was added to the *E. coli* pellet, and 100 µL of the mixture was spotted seven times onto a BHIS plate. The *E. coli* and *C. difficile* mixture was incubated for 13-18 hr after which the resulting growth was scraped from the plate into 1 mL PBS. One hundred microliters aliquots of the suspension were spread onto five BHIS plates containing 10 µg/mL thiamphenicol, 50 µg/mL kanamycin, and 8 µg/mL cefoxitin. The plates were incubated for 3-4 days at 37˚C, and transconjugants were passaged onto BHIS plates containing 15 µg/mL thiamphenicol, 50 µg/mL kanamycin, 8 µg/mL cefoxitin, and 5 µg/mL uracil. After selecting for the fastest growing colonies over 2-3 passages, single colonies were re-struck onto CDMM containing 2 mg/mL 5-FOA and 5 µg/mL uracil. FOA-resistant colonies that arose were patched onto CDMM containing FOA and uracil, and colony PCR was performed to identify clones harboring the desired deletions. Primer pairs #2009 and 2010, #2021 and 2022, and #1867 and

1868 were used to screen for deletions of *gerG*, *sleC*, and *spo0A*, respectively. The construction of the Δ*cspBAC* strain will be described more in detail in a future manuscript. All 630Δ*erm*Δ*pyrE* mutant strains were complemented with *pyrE* in the *pyrE* locus as described in the next section.

***C. difficile* complementation**

HB101/pRK24 donor strains carrying the appropriate complementation construct were grown in LB containing ampicillin (50 µg/mL) and chloramphenicol (20 µg/mL) at 37˚C, 225 rpm, under aerobic conditions, for 6 hrs. For pMTL83151-based conjugations, *C. difficile* recipient strains were conjugated as described previously (7, 8). For complementation in the *pyrE* locus using pMTL-YN1C constructs, a similar conjugation procedure was followed. Transconjugants were then re-struck onto CDMM and incubated for 2-4 days. Colonies that had restored the *pyrE* locus by virtue of their ability to grow on CDMM were re-struck onto CDMM before further characterization. At least two independent clones from each complementation strain were phenotypically characterized.

Artificial Germination.

Approximately 1 x 10^7 spores were pelleted and resuspended in 250 mM thioglycollate (artificial germination) or PBS (taurocholate-mediated germination) and processed as previously described (7). Ten microliters (10%) of the spore samples were plated on either BHIS or BHIS(TA) plates.

Antibody production.

The anti-CotA (CD1613), anti-GerG, and GerG(Δ1-7) antibodies used in this study were raised against CotA-His_6, GerG-His_6 and GerG(Δ1-7)-His_6, respectively, in rabbits by Cocalico Biologicals (Reamstown, PA). The His_6-tagged GerG variants and CotA were purified from *E. coli* strains #1084, 1625, and #852 using Ni^{2+}-affinity resin as previously described (9).

Western blot analyses.

C. *difficile* cell pellets were processed as previously described (4, 8). Samples were resolved by SDS-PAGE and transferred to Millipore Immobilon-FL membrane. The membranes were blocked in Odyssey Blocking Buffer. Rabbit polyclonal anti-GerG, anti-GerG(Δ1-7), anti-CspA antibody (a generous gift from Joseph Sorg), and anti-CotA antibodies were used at a 1:1000 dilution; the anti-CspB antibody was used at a 1:2500 dilution (9); and the anti-SleC (9) antibody was used at a 1:7000 dilution. CotA was used as a loading control for purified spores instead of SpoIVA as we have previously reported (7, 10, 11), since variation in the detection of SpoIVA between different spore preparations was observed (data not shown). The polyclonal mouse anti-SpoIVA (12) and anti-Spo0A (11) antibodies were used at a 1:2500 dilution. Infrared dye-conjugated secondary antibodies were used at 1:20,000 dilutions. The Odyssey LiCor CLx was used to detect secondary antibody infrared fluorescence emissions.

Accession numbers for GerG homologs in FIG 6.

Strains M120 (WP_003421789) and M68 (WP_003432566) encode an additional Asn-rich repeat relative to the 7 encoded by the other strains: 630(YP_001086780), CD42 (EQE58799), CD196 (WP_009888173), R20291 (CBE02008), and CD160 (WP_021382878).
