# Supplementary material for: A Clostridium difficile-Specific, Gel-Forming Protein Required for Optimal Spore Germination
Source: mBio. 2017 Jan 17;8(1):e02085-16. doi: 10.1128/mBio.02085-16 (PMC5241399; doi:10.1128/mBio.02085-16)
Supplement: FIG S8 [file mbo002173148sf8.pdf]

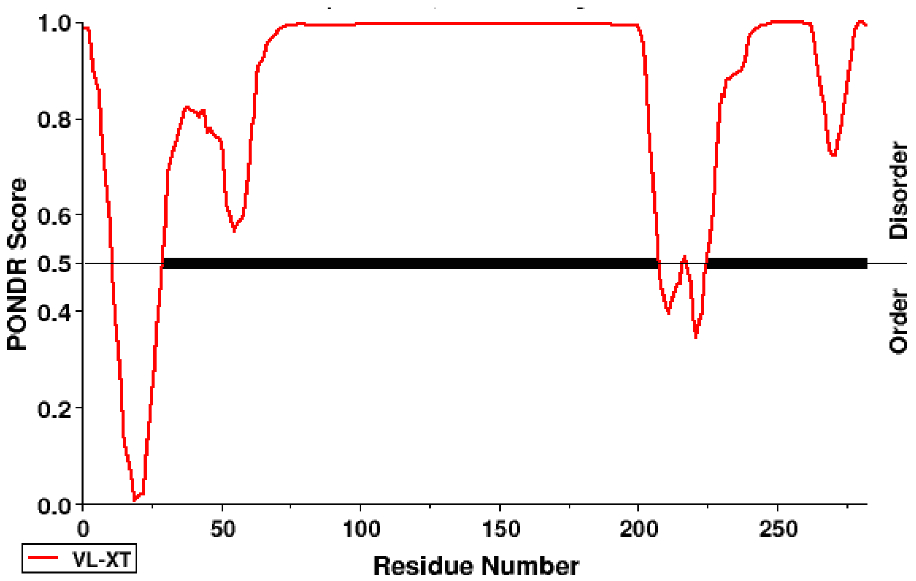


```
1      MSNKKKKDLD TSYMPYLDPA NYADYITNEE IPQDELRNDP QISNGYTKIP
VLXT   DDDDDDDDDD                     DD DDDDDDDDDD DDDDDDDDDD

51     NNQNINNLYP NNVNHYPKSS NSPNNLPNTN NTSGNMNYNT SATNNMPNNM
VLXT   DDDDDDDDDD DDDDDDDDDD DDDDDDDDDD DDDDDDDDDD DDDDDDDDDD

101    NNNTGMPNNM SNNMNSNMGM PNNMSNNMNS NMGMPNNMPN NMNSNMGMPN
VLXT   DDDDDDDDDD DDDDDDDDDD DDDDDDDDDD DDDDDDDDDD DDDDDDDDDD

151    NVPNNMNSNM GMPNNMSNNM NSNMGMPNNM SGNMNNNMYG NTGMPNNMNS
VLXT   DDDDDDDDDD DDDDDDDDDD DDDDDDDDDD DDDDDDDDDD DDDDDDDDDD

201    NMGYNMNNTM GIPNFSCNQN MPPNVLMMIP GVICHNTMQG MPVVMPSTMP
VLXT   DDDDDDD          D        DDDDDD DDDDDDDDDD DDDDDDDDDD

251    PNIYPTPYGS SNMSIQGIPQ ATNIEEFDEE EM
VLXT   DDDDDDDDDD DDDDDDDDDD DDDDDDDDDD DD
```

FIG S8. PONDR plot analysis of GerG. The heavy horizontal black bars indicate regions of predicted disorder as determined by PONDR using the VL-XT algorithm. The exact residues predicted to be disordered are indicated by a blue “D.”
